# Artificial intelligence-based digital pathology for the detection and quantification of soil-transmitted helminths eggs

**DOI:** 10.1371/journal.pntd.0012492

**Published:** 2024-09-30

**Authors:** Nancy Cure-Bolt, Fernando Perez, Lindsay A. Broadfield, Bruno Levecke, Peter Hu, John Oleynick, María Beltrán, Peter Ward, Lieven Stuyver

**Affiliations:** 1 Janssen Research & Development, LLC, Titusville, New Jersey, United States of America; 2 INMED, Andes Lima, Perú; 3 Enaiblers AB, Uppsala, Sweden; 4 Department of Translational Physiology, Infectiology and Public Health, Ghent University, Merelbeke, Belgium; 5 Janssen Research & Development, LLC, Raritan, New Jersey, United States of America; 6 Janssen Research & Development, LLC, Springhouse, Pennsylvania, United States of America; 7 Biologist, Independent Researcher and Consultant, Lima, Perú; 8 Global Public Health R&D, Janssen Pharmaceutica NV, Beerse, Belgium; Natural History Museum, UNITED KINGDOM OF GREAT BRITAIN AND NORTHERN IRELAND

## Abstract

**Background:**

Conventional microscopy of Kato-Katz (KK1.0) thick smears, the primary method for diagnosing soil-transmitted helminth (STH) infections, has limited sensitivity and is error-prone. Artificial intelligence-based digital pathology (AI-DP) may overcome the constraints of traditional microscopy-based diagnostics. This study in Ucayali, a remote Amazonian region of Peru, compares the performance of AI-DP-based Kato-Katz (KK2.0) method to KK1.0 at diagnosing STH infections in school-aged children (SAC).

**Methods:**

In this prospective, non-interventional study, 510 stool samples from SAC (aged 5–14 years) were analyzed using KK1.0, KK2.0, and tube spontaneous sedimentation technique (TSET). KK1.0 and KK2.0 slides were evaluated at 30-minute and 24-hour timepoints for detection of *Ascaris lumbricoides*, *Trichuris trichiura*, and hookworms (at 30-minute only). Diagnostic performance was assessed by measuring STH eggs per gram of stool (EPG), sensitivity of methods, and agreement between the methods.

**Results:**

KK2.0 detected more *A*. *lumbricoides* positive samples than KK1.0, with detection rates for *T*. *trichiura* and hookworms being comparable. At 30-minutes, 37.6%, 23.0%, and 2.6% of the samples tested positive based on KK1.0 for *A*. *lumbricoides*, *T*. *trichiura*, and hookworms, while this was 49.8%, 24.4%, and 1.9% for KK2.0. At 24-hours, 37.1% and 27.1% of the samples tested positive based on KK1.0 for *A*. *lumbricoides* and *T*. *trichiura*, while this was 45.8% and 24.1% for KK2.0. Mean EPG between KK2.0 and KK1.0 were not statistically different across STH species and timepoints, except for *T*. *trichiura* at 24-hours (higher mean EPG for KK1.0, *p* = 0.036). When considering infection intensity levels, KK2.0 identified 10% more of the total population as low-infection intensity samples of *A*. *lumbricoides* than KK1.0 (*p ≤* 0.001, both timepoints) and similar to KK1.0 for *T*. *trichiura* and hookworms. Varying agreement existed between KK1.0 and KK2.0 in detecting STH eggs (*A*. *lumbricoides*: moderate; *T*. *trichiura*: substantial; hookworms: slight). However, these findings should be interpreted carefully as there are certain limitations that may have impacted the results of this study.

**Conclusions:**

This study demonstrates the potential of the AI-DP-based method for STH diagnosis. While similar to KK1.0, the AI-DP-based method outperforms it in certain aspects. These findings underscore the potential of advancing the AI-DP KK2.0 prototype for dependable STH diagnosis and furthering the development of automated digital microscopes in accordance with WHO guidelines for STH diagnosis.

## Author summary

In tropical and sub-tropical regions with limited access to clean potable water, hygiene, and sanitation, infections with intestinal worms (e.g. giant roundworm, whipworm, and hookworms) are widespread, primarily affecting school-aged children (SAC). These infections lead to malnutrition, anemia, and developmental issues. The World Health Organization (WHO) introduced a strategy in 2020 to control intestinal worm infections, emphasizing the need for accurate diagnostic tests. The current recommended method, Kato-Katz (KK1.0), though cost-effective, has limitations in terms of reproducibility and sensitivity. To address this, an artificial intelligence digital pathology (AI-DP)-based Kato-Katz method (KK2.0) was developed to enhance diagnostic accuracy. This study in Ucayali (Amazonian region of Peru) evaluated KK2.0’s performance compared to KK1.0 in detecting intestinal worm infections among SAC. Stool samples from 510 SAC (5–14 years) were collected and analyzed using KK1.0 and KK2.0. KK2.0 demonstrated comparable diagnostic performance to KK1.0, with higher sensitivity for low-intensity giant roundworm infections. Prevalence rates differed between KK2.0 and KK1.0, indicating KK2.0’s potential to identify additional infections. Overall, the findings suggest KK2.0’s effectiveness in diagnosing STH infections, highlighting its potential to improve surveillance and treatment programs. Further research should address technical issues and assess KK2.0 in diverse settings, advancing its application for neglected tropical disease diagnosis.

## Introduction

Soil-transmitted helminths (STHs), i.e., *Ascaris lumbricoides*, *Trichuris trichiura*, hookworm (*Necator americanus* and *Ancylostoma duodenale*), and *Strongyloides stercoralis* infections are prevalent in tropical and sub-tropical regions that provide a conducive environment with limited access to clean potable water, inadequate sanitation, poor hygiene, and malnutrition resulting from poverty [1,2]. Globally, over 1.5 billion people, primarily school-aged children (SAC; 5–14 years), are estimated to be infected with STHs. Currently, ~654 million SAC and ~260 million pre-SAC (1–4 years) worldwide are at risk of STH infections [2]. These infections have a significant impact on public health, leading to malnutrition, anemia, and impairment in physical, intellectual, and cognitive development particularly in chronic cases among SAC. This emphasizes the urgent need for effective control measures [1–3].

The World Health Organization (WHO) approved a new STH infection control strategy in 2020, aiming to reduce the prevalence of moderate-to-heavy intensity infections among SAC and pre-SAC to <2% by 2030 [4]. To effectively gauge the success of the STH control program, it is crucial to utilize robust, sensitive, highly specific, and reliable diagnostic tests alongside prevention and treatment strategies [5,6].

Various diagnostic techniques have been utilized for the laboratory diagnosis of STHs, including but not limited to both microscopy-based methods (e.g., direct wet mount, the Kato-Katz method [KK]; henceforth designated as KK1.0]), tube spontaneous sedimentation technique (TSET), McMaster, Mini-FLOTAC, and formol-ether concentration, and immunodiagnostic-based methods (e.g., ELISA) and DNA-based methods (e.g., quantitative polymerase chain reaction [qPCR] and loop-mediated isothermal amplification). However, their sensitivity, cost, simplicity, and practicability vary [7–9].

Despite numerous technological advances in the diagnosis of STHs, applying microscopy methods on stool remains the reference standard for diagnosing these infections [10], due to their simplicity, ease-of-use in the field, and affordability. Currently, the simple KK1.0 is the WHO-recommended method for detecting STHs. Also, KK1.0 is a cost-effective method as kits are low-cost, compact, and can be washed and reused. [11–13]. KK1.0 facilitates the measurement of infection by counting the eggs in a specified volume of stool and by multiplying the counts with a factor that allows the conversion to eggs per gram of stool (EPG). Fecal egg counts reported as EPG are then used as a proxy for infection intensity and serve as an indirect measure of STH burden and morbidity. Importantly, the accurate measurement of prevalence of any STH of any intensity is crucial in making judgements to define the frequency of mass drug administration (MDA) programs in STH management in endemic regions [14].

In previous studies, KK1.0 has demonstrated sensitivity comparable to that of other microscopy-based or molecular methods and has performed well in identifying moderate-to-heavy intensity STH infections [11,15,16]. However, it is not deemed ideal due to its limited reproducibility, an error-prone manual read-out, low sensitivity for low-intensity infections, and the potential for greater time between stool sample collection and laboratory processing (in cases where KK1.0 testing is conducted in a centralized lab, as is the case in Peru) [11,17]. Therefore, adopting more sensitive, specific, and cost-effective diagnostic methods that are designed to align with the WHO target product profiles (TPPs; specificity: ≥94%, sensitivity: ≥60%), is crucial for effectively controlling and eliminating STH-attributable morbidity in endemic regions [18–20].

In this context, emerging diagnostic artificial intelligence-based digital pathology (AI-DP) could prove beneficial for overcoming the constraints of traditional microscopy-based diagnostics and advancing towards the target of WHO 2030 [4,21–25]. AI-DP has the potential to enhance diagnostic accuracy, reduce operational costs by boosting throughput and decreasing labor needs, and automate data collection, processing, and reporting [24,25]. Furthermore, AI-DP principles imply the ability of detecting, categorizing, and quantifying egg-like features, facilitating the classification of STH infection intensity over the whole spectrum, from no infection to low, moderate, and heavy intensity infections [25]. A KK based AI-DP prototype (henceforth designated as KK2.0), developed by Ward et al. [26], aims to increase the clinical sensitivity and specificity of KK1.0 by automating the scanning and detection of STH eggs in stool smears prepared using KK1.0. The KK2.0 system comprises a whole slide imaging (WSI) scanner, an AI model, and a data reporting system, with analysis based on numerous microscopic field-of-views per smear. The KK2.0 system possesses the capability to analyze egg-like features in the field independently of internet infrastructure. Additionally, a telemedicine approach, involving the evaluation of data from a distant central facility, has been implemented. This prototype has been tested in field studies in other endemic countries and image databases have been built, however, its clinical sensitivity and specificity had not been assessed until this study [26].

In Latin America (LATAM) and the Caribbean, at least 35.4 million SAC and 13.9 million pre-SAC are estimated to be at risk of infection [27]. In Peru, the prevalence of STHs among SAC varies widely, ranging from 1.6% to 78.9% [28]. Despite numerous control measures and deworming, STH infections remain a significant public health concern, especially in the Amazonia region where environmental conditions favor STH development [29–32]. In this region, the qualitative TSET is used as a standard method for monitoring the STH prevalence rather than the widely used KK1.0 [9,33]. TSET, initially introduced by Tello in Peru, is renowned for its simplicity, cost-effectiveness, and efficiency in diagnosing STH infections. During this study, in accordance with the Ethics Committee regulations, TSET was included only to provide locally accepted results, prompting parents of participating children to seek medical care if STH infection was detected. Considering the lack of sample volume standardization, the TSET method is unable to provide an EPG or equivalent statistic, thereby hindering the comprehensive understanding of the actual burden and impact of STH infections in the region.

Different diagnostic methods may impact the assessment of the true burden of STH infections and decisions regarding medication use in prevention and control programs. Therefore, it is crucial to validate the KK2.0 approach, ultimately strengthening the prospects for future STH programs [33].

Hence, this prospective, non-interventional study was conducted to evaluate the KK2.0 method and compare its diagnostic performance with KK1.0 at detecting *A*. *lumbricoides*, *T*. *trichiura*, and hookworms among SAC residing in selected areas of Ucayali, a remote Amazonian region of Peru. Furthermore, this study is an early step towards strengthening the existing STH program surveillance by providing a tool that improves the sensitivity of current testing methods and timely access to high-quality data.

## Materials and methods

### Ethics statement

The study protocol (**S1 Info**) was approved by the National Ethical Committee, part of the Peruvian National Institute of Health (N°418-2022/CIEI-IIN). The study was conducted in accordance with the ethical principles originating in the Declaration of Helsinki and the International Conference on Harmonisation, Good Clinical Practice guidelines, applicable regulatory requirements, and in compliance with the protocol. All parents or guardians provided written informed consent for their children to participate; child (ages 5 to 7 years) and adolescent (ages 8 to 14 years) assent was obtained for data and sample collection.

### Study population

SAC between 5 and 14 years of age, who were reported to be healthy, able to provide a sufficient stool sample (~10 grams), and spoke Spanish were enrolled; non-Spanish speakers were not considered as consent form was in Spanish and the research and field teams comprised of only Spanish speakers. Those with active diarrhea (≥3 loose/liquid stools/day) at baseline, having an acute or severe medical condition, or who had received anthelmintic treatment within 90 days prior to study entry were excluded.

### Study design

This was a prospective, non-interventional field study conducted in selected STH endemic areas of Ucayali, a remote Amazonian region of Peru, from August 2022 to October 2023. At least two weeks before collecting samples (visit 1), field workers surveyed the chosen area to locate households with SAC. The day before sample production and collection visit (visit 2), the field workers visited the homes of SAC within the study population. They explained the study to parents or guardians and children, presented educational flip books explaining the study, and obtained their consent and assent. A clean, leak-proof stool sample container was provided to each study participant; a fresh stool sample (~10 grams) was to be provided on a prespecified day and time (visit 3).

### Sample processing and analysis

**Fig 1:**
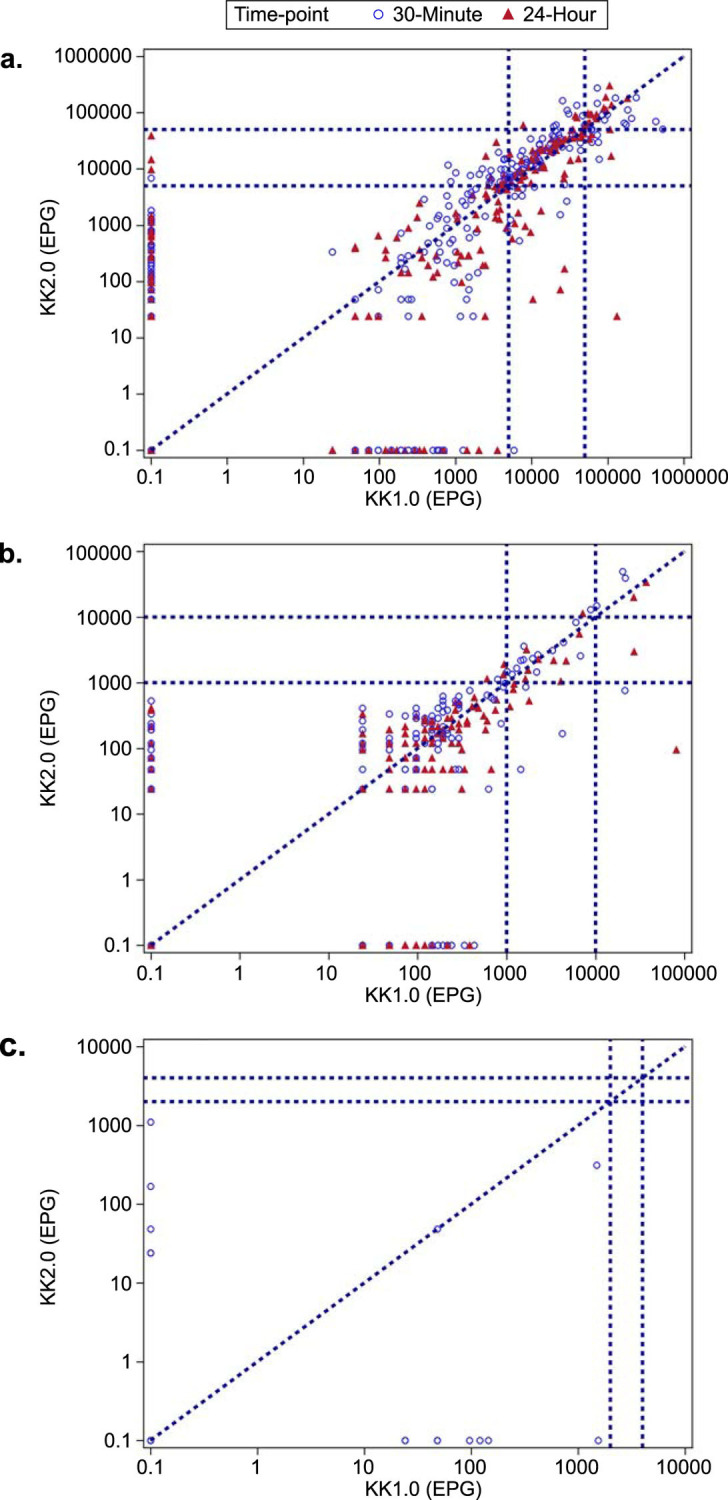
Study design. AI, artificial intelligence, EPG, eggs per gram, KK1.0, traditional Kato-Katz method; KK2.0, artificial intelligence digital pathology Kato- Katz method; T, time. The study design is explained on pages 8 to 10.

**Fig 1** depicts the process of sample processing and analysis.

Each stool sample was transferred to the local laboratory in an ice-filled container within 4±1 hours of collection. The samples were manually homogenized and processed within 1 hour of receipt to detect the presence of *A*. *lumbricoides*, *T*. *trichiura*, and hookworm (*N*. *americanus* and *A*. *duodenale*) eggs. The samples were assessed by the TSET, KK1.0, and KK2.0 methods. In the TSET assay, a slide was prepared from each sample as per the standard operating procedure [9]. The presence of STH eggs and larvae was examined under a microscope with 10x and 40x objectives within 30±10 minutes.

For the KK methods, each sample was processed into 2 thick smear slides (slide A and slide B), which were prepared by separate laboratory technicians using the previously reported standard operating procedures [13]. Slides were randomly assigned to be evaluated either by KK1.0 or KK2.0 first, preventing any order bias in the results. Both slides were analyzed within 30±10 minutes (henceforth designated as 30-minute) following slide preparation, to count hookworm eggs [11, 34] as they would clear and disappear from stool samples within this window of time. Slide A was assessed by the KK1.0 method to manually count *A*. *lumbricoides*, *T*. *trichiura*, hookworm eggs, and any other recognizable parasites. Slide B was evaluated using the KK2.0 method for automated egg counting. The slide was placed in the AI-DP slide scanner, which automatically performed WSI. An on-board AI model processed the images and identified egg-like features for verification (details in **S2 Info**) [26].

Slide B was refrigerated overnight to clear it of sediments and debris and facilitate easy identification of *A*. *lumbricoides* and *T*. *trichiura* eggs at the 24±4-hour timepoint (henceforth designated as 24-hour) using KK1.0 and KK2.0.

Data obtained from reading slides by the KK1.0 method were converted to EPG and uploaded to the study database. The images of all slides scanned into the AI-DP tool (KK2.0) on day 1 and day 2 were first processed by the AI model locally on the device, before being uploaded to the cloud database, allowing remote verifiers to review the egg-like features in the EggInspector application. The verifiers inspected only the suspected eggs as determined by the AI, and not the whole slide, as a result, false negatives could not be quantified. The images were validated by an expert parasitologist in Peru; subsequent cloud-based verifications at the second and third levels were performed by an expert parasitologist in Uganda and Ethiopia. Validated results were transferred to the study database.

### Data quality control

Reliability of the study findings was ensured by randomly re-examining 10% of the KK1.0 and TSET slides by an independent laboratory technician. For the KK2.0 method, 100% of the slides were verified by an expert parasitologist (recounted eggs).

### Study endpoints

The primary objective of this study was to compare the diagnostic performance of KK2.0 with KK1.0 for detecting eggs of *A*. *lumbricoides*, *T*. *trichiura*, and hookworms (*N*. *americanus* and *A*. *duodenale*). Diagnostic performance was determined by assessing the following clinical outcomes for the three STHs at the 30-minute and 24-hour timepoints after slide preparation: (i) EPG of KK1.0 and KK2.0; (ii) sensitivity of KK1.0 and KK2.0; (iii) agreement between KK1.0 and KK2.0 in identifying infection; (iv) agreement between KK1.0 and KK2.0 in classifying the infection intensity; and (v) SAC infection intensity of KK1.0 and KK2.0. Additionally, the prevalence of any STH (aforementioned species) in the selected endemic region was assessed.

### Statistical analysis

This study was designed to generate descriptive data for informative purposes. No formal statistical hypothesis was tested; hence, no formal sample size calculations were performed. The planned sample size of 500 SAC was based on pragmatic considerations.

Descriptive statistics were used to summarize all continuous variables (EPG), while frequencies and percentages were used to summarize all categorical variables. Two categorical variables were included, derived based on egg count or EPG: the status of SAC categorized as either with or without eggs, from which sensitivity was derived; and the status of SAC with low, moderate, or heavy infection intensity based on EPG. Summary and analysis were provided by the diagnostic method, STH species, and timepoints (30-minute and 24-hour).

As the TSET method is unable to produce EPG, the intensity of each STH species was derived for KK1.0 and KK2.0 only. As no standard for egg counts was available for this study, a diagnostic pseudo standard was created for calculating sensitivity. However, it falls short of the ideal of 100% sensitivity and specificity that a true reference standard would provide. A sample was considered truly positive if any diagnostic method returned a positive reading at any point throughout the investigation. Formally, the set of truly positive samples (denoted as Standard_+_) for an STH species was defined as the union of the sets of positive samples for each diagnostic method and timepoint:

Standard_+_ = KK1.0_+,30m_ ∪ KK2.0_+,30m_ ∪ KK1.0_+,24h_ ∪ KK2.0_+,24h_ ∪ TSET_+_

where,

KK1.0_+,30m_ and KK2.0_+,30m_are set of positive samples based on KK1.0 and KK2.0 at 30 minutes, respectively

KK1.0_+,24h_ and KK2.0_+,24h_are set of positive samples based on KK1.0 and KK2.0 at 24 hours, respectively

TSET_+_ is set of positive samples based on TSET, performed only once during the study

For EPG and sensitivity comparisons, test results with *p* ≤0.05 were considered statistically significant.

## Analysis of endpoints

### Primary endpoint

Mean EPG was summarized by the diagnostic method, STH species, and timepoint.

Sensitivity for KK1.0 at 30 minutes was calculated as:

$$=\frac{\text{N}\left({\text{KK}}^{2}.0_{+,30\text{m}}\right)}{\text{N}\left({\text{Standard}}_{+}\right)}$$

where, N(KK1.0_+,30m_) is the number of positive samples based on KK1.0 method at 30 minutes, and N(Standard_+_) is the number of truly positive samples, as defined above.

Sensitivity for other diagnostic methods and other timepoints was calculated similarly. A permutation test was used to compare EPG and sensitivity between different diagnostic methods based on the absolute difference. Subsequently, the agreement in assessing infection between KK1.0 and KK2.0 was evaluated by classifying samples into egg/no egg, and the Kappa statistic was calculated. Agreement in assessing the infection intensity between KK1.0 and KK2.0 was further evaluated by classifying samples into categories of no, low, moderate, and heavy infection based on EPG, and the weighted Kappa statistic was calculated. In general, the Kappa statistics values ≤0 indicate no agreement, 0.01–0.20 signify none-to-slight, 0.21–0.40 indicate fair agreement, 0.41–0.60 suggest moderate agreement, 0.61–0.80 reflect substantial agreement, and 0.81–1.00 represent almost perfect agreement [35].

### Other endpoints

The STH prevalence was defined as the percentage of SAC with positive egg counts and summarized based on the diagnostic method and the STH species. If a SAC tested positive for a STH species at any point during the study, he or she was deemed infected. TSET qualitative readings (1+, 2+, 3+) were also summarized.

## Results

### Participant disposition and demographics

A total of 510 SAC enrolled in this study and provided stool samples. Mean (standard deviation (SD)) age was 9.2 (2.80) years. Of 510 SACs enrolled, the distribution of girls (225 [50%]) and boys (225 [50%]) was equal. Most participants (66%) were in primary school (**S1 Table**). For TSET, 508 (99.6%) samples were assessed only at 30-minute timepoint. For KK1.0, 508 (99.6%) samples were assessed at the 30-minute timepoint and 502 (98.4%) samples at the 24-hour timepoint. For KK2.0, 484 (94.9%) samples were assessed at the 30-minute timepoint, and 465 (91.2%) samples were assessed at the 24-hour timepoint. Most of the samples lost for KK2.0 were due to either loss of electricity, WSI malfunctions, or minor human errors. Issues were logged during the study to continue improvement in the KK2.0 development.

## Primary endpoints

### Eggs per gram

Using KK2.0, a higher proportion of positive samples for *A*. *lumbricoides* was identified compared to KK1.0, whereas the proportions for *T*. *trichiura* and hookworms were comparable. At the 30-minute timepoint with KK1.0, 191/508 (37.6%) were positive for *A*. *lumbricoides*, 117/508 (23.0%) for *T*. *trichiura*, and 13/506 (2.6%) for hookworms. With KK2.0, 241/484 (49.8%) were positive for *A*. *lumbricoides*, 118/484 (24.4%) for *T*. *trichiura*, and 9/472 (1.9%) for hookworms. At the 24-hour timepoint, using KK1.0, 186/502 (37.1%) samples were positive for *A*. *lumbricoides*, and 136/502 (27.1%) for *T*. *trichiura*. With KK2.0, 213/465 (45.8%) were positive for *A*. *lumbricoides* and 112/465 (24.1%) for *T*. *trichiura* (**Tables 1 and S2**).

**Fig 2:**
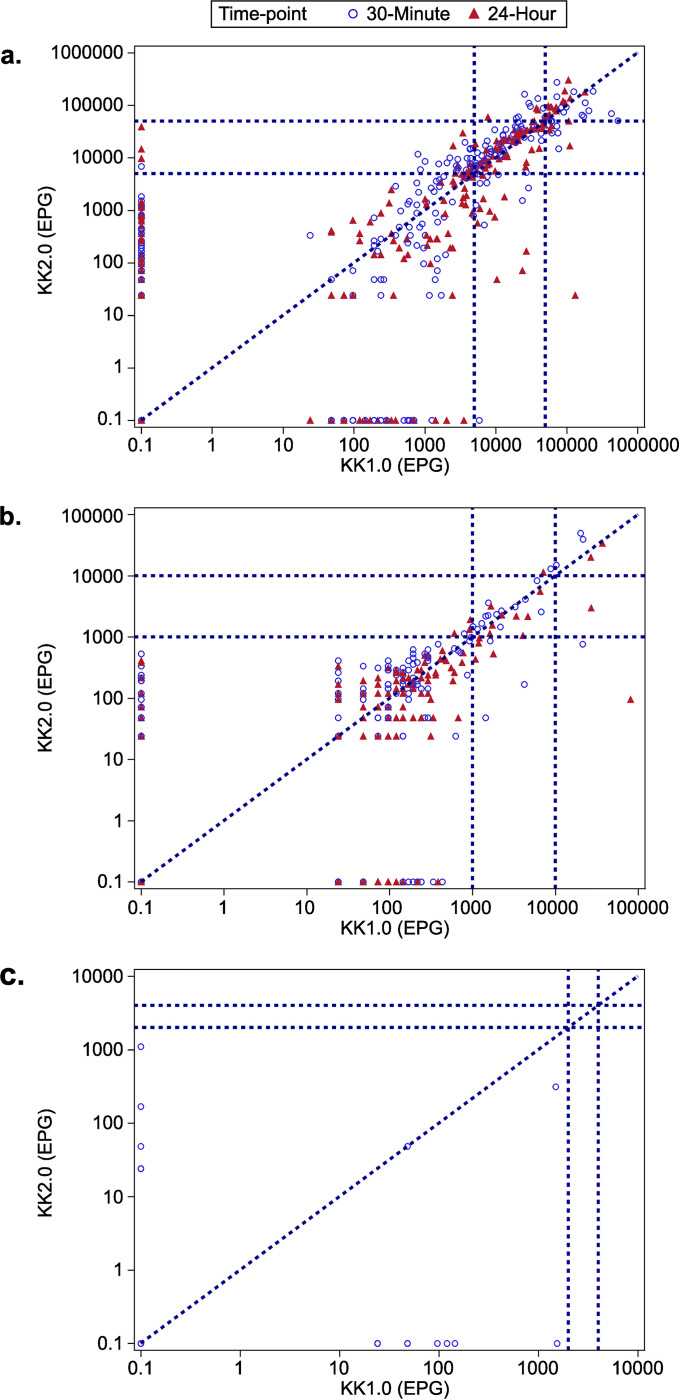
Scatterplot of EPG of *Ascaris lumbricoides* (a), *Trichuris trichiura* (b), and hookworms (c) for KK1.0 and KK2.0 at 30-minute and 24-hour timepoints. For hookworm, there were very few sample points, considering the majority of samples were negative for this STH. EPG, eggs per gram; KK1.0, Kato-Katz method; KK2.0, artificial intelligence digital pathology Kato-Katz method. *A*. *lumbricoides*: low: 1 to <5,000 EPG; moderate: 5,000 to 49,999 EPG; heavy: ≥50,000 EPG. *T*. *trichiura*: low: 1 to <1,000 EPG; moderate: 1,000 to 9,999 EPG; heavy: ≥10,000 EPG. Hookworms: low: 1 to <2,000 EPG; moderate: 2,000 to 3,999 EPG; heavy: ≥4,000 EPG EPG is presented on a log_10_ scale axis. Samples with an EPG of 0 are given a value of 0.1 to allow for presentation on a log axis. Results from both 30-minutes and 24-hours are presented for each participant (for hookworms, only at 30 minutes). Hookworm samples are excluded from analysis if the time between slide preparation end time and slide reading start time is outside the range of 20 to 80 minutes (inclusive).

No significant difference in the mean EPG was found between the two diagnostic methods for the three STHs, except for *T*. *trichiura* at the 24-hour timepoint (KK1.0 reported significantly higher mean EPG than KK2.0 [*p* = 0.036; **Table 1** and **Fig 2**]). For both methods, mean EPG of *A*. *lumbricoides* was higher at the 30-minute timepoint than the 24-hour timepoint; a similar pattern was observed for KK2.0 for *T*. *trichiura*, although mean EPG was higher at the 24-hour timepoint than the 30-minute timepoint for KK1.0.

**Table 1:** EPG by diagnostic method, STH species, and timepoint.

STH Species	Timepoint	KK1.0			KK2.0			*p*-value
		N	Mean (SD)	95% CI	N	Mean (SD)	95% CI	
*Ascaris lumbricoides*	30-minute	508	8,720.4 (38,609.6)	5,696.2, 12,391.1	484	7,685.8 (25,492.9)	5,542.4; 10,091.8	0.513
	24-hour	502	6,849.2 (19,932.6)	5,178.6, 8,646.1	465	7,534.5 (25,390.1)	5,377.0; 9,969.2	0.355
*Trichuris trichiura*	30-minute	508	280.4 (1,796.5)	143.9, 451.8	484	368.6 (3,056.7)	141.7; 675.4	0.296
	24-hour	502	519.3 (4,395.4)	200.0, 961.0	465	237.0 (1,941.3)	92.8; 438.9	0.036
Hookworms	30-minute	506	7.3 (95.5)	0.9, 16.5	472	3.8 (53.4)	0.4; 9.5	0.399

95% confidence interval (95% CI) is based on bootstrapping. A permutation test was employed for comparison of eggs per gram of stool (EPG) between different diagnosis methods based on absolute mean difference. Hookworm samples are excluded from analysis if the time between slide preparation end time and slide reading start time is outside the range of 20 to 80 minutes (inclusive).

KK1.0, traditional Kato-Katz method; KK2.0, artificial intelligence digital pathology Kato-Katz method; SD, standard deviation; STH, soil-transmitted helminth.

### Sensitivity of KK1.0 and KK2.0

The sensitivity of KK2.0 was higher than KK1.0 when detecting *A*. *lumbricoides* at both timepoints, and the difference was significant (*p* <0.001). The sensitivity of KK1.0 and KK2.0 was similar for *T*. *trichiura* at both timepoints, and at the 30-minute timepoint for hookworms (**Table 2**).

**Table 2:** Sensitivity by diagnostic method, STH Species, and timepoint (Analysis set: evaluable).

	30-minute			24-hour		
	KK 1.0	KK 2.0	*p*-value	KK 1.0	KK 2.0	*p*-value
***Ascaris lumbricoides***						
n/N	191/337	241/315	0.000	186/333	213/301	0.000
Estimate	0.57	0.77		0.56	0.71	
95% CI	(0.51, 0.62)	(0.72, 0.81)		(0.51, 0.61)	(0.66, 0.76)	
***Trichuris trichiura***						
n/N	117/169	118/162	0.352	136/167	112/152	0.054
Estimate	0.69	0.73		0.81	0.74	
95% CI	(0.62, 0.76)	(0.66, 0.80)		(0.75, 0.87)	(0.66, 0.81)	
**Hookworms**						
n/N	13/26	9/26	0.229			
Estimate	0.50	0.35				
95% CI	(0.30, 0.70)	(0.17, 0.54)				

95% confidence interval (95% CI) is based on bootstrapping.

A permutation test was employed for comparison of sensitivity between different diagnosis methods based on absolute difference.

Hookworm samples were excluded from analysis if the time between slide preparation end time and slide reading start time was outside the range of 20 to 80 minutes (inclusive).

N is the number of samples with a positive egg count for that egg type according to the pseudo standard. A sample was considered truly positive if any diagnostic method comes back with a positive reading at any time of the study. For each egg type, if a sample was missing at a specific timepoint for a diagnostic method, that sample is excluded in entirety from the pseudo gold standard for that timepoint and diagnostic method.

KK1.0, traditional Kato-Katz method; KK2.0, artificial intelligence digital pathology- Kato-Katz method; n, number of positive samples; STH, soil-transmitted helminth.

### Agreement in KK1.0 and KK2.0 results

To assess the agreement between KK1.0 and KK2.0, a sample was classified as either positive (egg) or negative (no egg) based on the egg count. At the 30-minute timepoint, 152 (31.4%) samples were identified as positive by both KK1.0 and KK2.0, 217 (44.8%) samples were identified as negatives by both KK1.0 and KK2.0. In other words, KK1.0 and KK2.0 agreed on 76.2% of samples; 89 (18.4%) samples were negative by KK1.0 but positive by KK2.0 for *A*. *lumbricoides*; while 26 (5.4%) samples were negative by KK2.0 but positive by KK1.0. A similar pattern was observed for the 24-hour timepoint. The Kappa statistics indicated a moderate level of agreement between KK1.0 and KK2.0 in identifying *A*. *lumbricoides* eggs. There was substantial agreement in identifying *T*. *trichiura* eggs between the two methods at both timepoints. At the 30-minute timepoint, KK1.0 and KK2.0 agreed on 91.9% of samples; 22 (4.5%) samples were negative by KK1.0 but positive by KK2.0 for *T*. *trichiura*, while 17 (3.5%) samples were negative by KK2.0 but positive by KK1.0. A similar pattern was observed for the 24-hour timepoint. There was slight agreement in identifying hookworm eggs at the 30-minute timepoint, with 95.7% agreement between KK1.0 and KK2.0 (**Table 3** and **Fig 2**).

For the 89 samples identified as negative by KK1.0 but positive by KK2.0 for *A*. *lumbricoides* at the 30-minute timepoint, most of them (n = 88) were classified as low-intensity infection by KK2.0. A similar pattern was observed for *A*. *lumbricoides* at the 24-hour timepoint (**Table 3** and **Fig 2**). At both timepoints, there was substantial agreement between KK1.0 and KK2.0 in classifying the infection intensity of *A*. *lumbricoides* and *T*. *trichiura*. There were no moderate or heavy infections for hookworms using either method; therefore, agreement in identifying infection intensity could not be calculated (**Table 3**).

**Table 3:** Agreement between diagnostic methods by egg type (Analysis set: evaluable).

**Timepoint**	**Egg Type**		**KK1.0**		**KK2.0**				**Kappa Statistics**
					**Egg, n (%)**		**No Egg, n (%)**		
**Any infection intensity**									
**30-minute**	*Ascaris lumbricoides*		Egg, n (%)		152 (31.4)		26 (5.4)		0.524
			No Egg, n (%)		89 (18.4)		217 (44.8)		
	*Trichuris trichiura*		Egg, n (%)		96 (19.8)		17 (3.5)		0.778
			No Egg, n (%)		22 (4.5)		349 (72.1)		
	Hookworms		Egg, n (%)		2 (0.4)		11 (2.3)		0.163
			No Egg, n (%)		7 (1.5)		450 (95.7)		
**24-hour**	*Ascaris lumbricoides*		Egg, n (%)		145 (31.2)		24 (5.2)		0.595
			No Egg, n (%)		68 (14.6)		228 (49.0)		
	*Trichuris trichiura*		Egg, n (%)		98 (21.1)		26 (5.6)		0.773
			No Egg, n (%)		14 (3.0)		327 (70.3)		
**Infection Intensity**									
**Timepoint**	**Egg Type**	**KK1.0**		**KK2.0**					**Weighted Kappa Statistic**
				**No Eggs** **n (%)**	**Low** **n (%)**	**Moderate** **n (%)**		**Heavy** **n (%)**	
**30-minute**	*Ascaris lumbricoides*	No Eggs, n (%)		217 (44.8)	88 (18.2)	1 (0.2)		0	0.623
		Low, n (%)		25 (5.2)	51 (10.5)	20 (4.1)		0	
		Moderate, n (%)		1 (0.2)	10 (2.1)	42 (8.7)		8 (1.7)	
		Heavy, n (%)		0	0	6 (1.2)		15 (3.1)	
	*Trichuris trichiura*	No Eggs, n (%)		349 (72.0)	22 (4.5)	0		0	0.792
		Low, n (%)		17 (3.5)	74 (15.3)	1 (0.2)		0	
		Moderate, n (%)		0	3 (0.6)	13 (2.7)		1 (0.2)	
		Heavy, n (%)		0	1 (0.2)	0		3 (0.6)	
	Hookworms	No Eggs, n (%)		450 (95.7)	7 (1.5%)	0		0	NC
		Low, n (%)		11 (2.3)	2 (0.4%)	0		0	
		Moderate, n (%)		0	0	0		0	
		Heavy, n (%)		0	0	0		0	
**24-hour**	*Ascaris lumbricoides*	No Eggs, n (%)		228 (49.0)	65 (14.0)	3 (0.6)		0	0.689
		Low, n (%)		24 (5.2)	50 (10.8)	10 (2.2)		0	
		Moderate, n (%)		0	10 (2.2)	50 (10.8)		6 (1.3)	
		Heavy, n (%)		0	1 (0.2)	3 (0.6)		15 (3.2)	
	*Trichuris trichiura*	No Eggs, n (%)		327 (70.3)	14 (3.0)	0		0	0.762
		Low, n (%)		26 (5.6)	78 (16.8)	3 (0.6)		0	
		Moderate, n (%)		0	4 (0.9)	8 (1.7)		1 (0.2)	
		Heavy, n (%)		0	1 (0.2)	1 (0.2)		2 (0.4)	

Only evaluable samples with readings from both KK1.0 and KK2.0 are included. Hookworm samples are excluded from analysis if the time between slide preparation end time and slide reading start time is outside the range of 20 to 80 minutes (inclusive).

Higher Kappa statistics value indicates stronger agreement between the two diagnostic methods.

*A*. *lumbricoides*: low: 1 to <5,000 EPG; moderate: 5,000 to 49,999 EPG; heavy: ≥50,000 EPG

*T*. *trichiura*: low: 1 to <1,000 EPG; moderate: 1,000 to 9,999 EPG; heavy: ≥10,000 EPG

Hookworms: low: 1 to <2,000 EPG; moderate: 2,000 to 3,999 EPG; heavy: ≥4,000 EPG

CI, confidence interval; EPG, eggs per gram of stool; KK1.0, traditional Kato-Katz method; KK2.0, artificial intelligence digital pathology Kato-Katz method; NC, not calculated.

### SAC infection intensity

The majority of difference in detecting infection intensity between KK1.0 and KK2.0 were noted in the low-intensity infection category for *A*. *lumbricoides*, where KK2.0 identified ~10% more samples (30-minute timepoint: 20.5% [KK1.0] *vs*. 30.8% [KK2.0]; 24-hour timepoint: 17.5% [KK1.0] *vs*. 27.1% [KK2.0]) (**Table 4**). This finding is consistent with the analysis of agreement in the infection intensity between KK1.0 and KK2.0. The distribution of the infection intensity by EPG (no eggs, low, moderate, and heavy) was similar for KK1.0 and KK2.0 at both timepoints for *T*. *trichiura* and at the 30-minute timepoint for hookworms.

**Table 4:** Summary of SAC intensity by diagnostic method and STH species, and timepoint.

	KK1.0	KK2.0
30-Minute	N = 508	N = 484
*Ascaris lumbricoides*, n (%)		
No Eggs	317 (62.4)	243 (50.2)
Low	104 (20.5)	149 (30.8)
Moderate	64 (12.6)	69 (14.3)
Heavy	23 (4.5)	23 (4.8)
*Trichuris trichiura*, n (%)		
No Eggs	391 (77.0)	366 (75.6)
Low	96 (18.9)	100 (20.7)
Moderate	17 (3.3)	14 (2.9)
Heavy	4 (0.8)	4 (0.8)
Hookworms, n (%)		
No Eggs	493 (97.0)	463 (95.7)
Low	13 (2.6)	9 (1.9)
24-Hour	N = 502	N = 465
*Ascaris lumbricoides*, n (%)		
No Eggs	316 (62.9)	252 (54.2)
Low	88 (17.5)	126 (27.1)
Moderate	76 (15.1)	66 (14.2)
Heavy	22 (4.4)	21 (4.5)
*Trichuris trichiura*, n (%)		
No Eggs	366 (72.9)	353 (75.9)
Low	116 (23.1)	97 (20.9)
Moderate	15 (3.0)	12 (2.6)
Heavy	5 (1.0)	3 (0.6)

Hookworm samples are excluded from analysis if the time between slide preparation end time and slide reading start time is outside the range of 20 to 80 minutes (inclusive).

*A*. *lumbricoides*: low: 1- = to <5,000 EPG; moderate: 5,000 to 49,999 EPG; heavy: ≥50,000 EPG

*T*. *trichiura*: low: 1 to <1,000 EPG; moderate: 1,000 to 9,999 EPG; heavy: ≥10,000 EPG

Hookworms: low: 1 to <2,000 EPG; moderate: 2,000 to 3,999 EPG; heavy: ≥4,000 EPG.

EPG, eggs per gram of stool; KK1.0, traditional Kato-Katz method; KK2.0, artificial intelligence digital pathology Kato-Katz method; SAC, school-aged children; STH, soil-transmitted helminth.

### Prevalence of STHs

KK2.0 identified a substantially higher prevalence of *A*. *lumbricoides* infection (300 [59.1%]) than KK1.0 (218 [42.9%]) and TSET (193 [38.0%]). KK1.0 identified slightly higher *T*. *trichiura* infection (153 [30.1%]) than KK2.0 (133 [26.2%]) and TSET (119 [23.4%]). The prevalence of hookworm infections was low for all three methods (KK1.0: 13 [2.6%]; KK2.0: 9 [1.8%]; TSET: 16 [3.1%]) (**Table 5**). If a participant tested positive at either timepoint, they were classified as positive and included in the calculation of infection prevalence.

**Table 5:** Prevalence of STH species by diagnostics methods.

STH Species	KK1.0 N = 508	KK2.0 N = 508	TSET N = 508
*Ascaris lumbricoides*, n (%)	218 (42.9)	300 (59.1)	193 (38.0)
*Trichuris trichiura*, n (%)	153 (30.1)	133 (26.2)	119 (23.4)
Hookworms, n (%)	13 (2.6)	9 (1.8)	16 (3.1)

KK1.0, traditional Kato-Katz method; KK2.0, artificial intelligence digital pathology Kato-Katz method; STH, soil-transmitted helminth; TSET, tube spontaneous sedimentation technique.

## Discussion

Effective STH control initiatives demand accurate, specific, sensitive, and cost-effective diagnostic techniques to ensure precise surveillance and treatment monitoring [5,6]. However, in endemic regions like Peru, the use of a qualitative diagnostic method results in the underdiagnosis and underreporting of STHs [6,15,33], specifically among SAC who bear a higher burden of STH infections and are the primary target of MDA campaigns [28,30,36]. Currently, most STH prevention programs rely on using the cost-effective KK1.0 method for population surveys, which has near-perfect specificity and limited sensitivity for low-intensity infections [11,17,21,22]. Although more sensitive and accurate molecular techniques like (q)PCR exist, their utilization for large-scale screening is unfeasible due to their high cost and the need for skilled personnel, especially in resource-limited settings [7]. Therefore, AI was implemented on KK1.0 to increase its clinical sensitivity, while maintaining near-perfect specificity.

In this study, the AI-DP prototype KK2.0, featuring automated sample scanning and fecal egg counting, demonstrated concordance to KK1.0 in identifying STH species. The performance of KK2.0 in determining the EPG count was comparable to KK1.0 at both 30-minute and 24-hour timepoints, except for the *T*. *trichiura* count at the 24-hour timepoint. Notably, KK1.0 exhibited considerably greater mean EPG for *T*. *trichiura* than KK2.0 at the 24-hour timepoint. Consistent with previously reported results [11], the mean EPG of only *T*. *trichiura* was higher at the 24-hour timepoint than the 30-minute timepoint using the KK1.0 method (30-minute: 280.4 EPG, 24-hour: 519.3 EPG). This increase in the *T*. *trichiura* EPG count could potentially be attributed to the natural variation in egg counting and reader interpretation differences [11]. This variation may have arisen because the slides were not randomly assigned to laboratory technicians and the technicians were not blinded. However, this potential impact observed with KK1.0 could have been eliminated with the KK2.0 method through the implementation of automated egg counting. It is also worth noting that a longer waiting period after smear preparation could enhance *T*. *trichiura* egg visibility [11], but this effect was disregarded since both KK1.0 and KK2.0 used the same slide (slide B) for detection. Future work should explore verification of all images in a scanned slide to establish a ground truth for egg counts at the two timepoints.

Interestingly, KK2.0 detected more *A*. *lumbricoides* infections than KK1.0, with this difference being particularly pronounced in the low-intensity infection category. This marks a crucial finding, especially when considering lower STH detection by KK1.0 in areas with low prevalence of STH [6,11,37]. It is important to highlight that the AI dataset for *A*. *lumbricoides* was more extensive before this study, potentially contributing to the observed outcomes in low-intensity infections [26]. Moreover, with the expansion of the AI dataset for other STHs, similar findings in low-intensity infections for those are anticipated.

A considerable number of low-intensity infections, which might go undiagnosed by KK1.0, can also contribute to and result in high prevalence settings. This necessitates additional rounds of MDA despite the low-intensity infections. Adhering to the KK2.0 results in this study would mean conducting two rounds of MDA annually in this region, implying that the children are currently undertreated. For *T*. *trichiura*, both diagnostic methods showed similar distribution of infection intensity. At the 30-minute timepoint, hookworm identification was low, precluding any conclusive interpretation. The protocol in the present study strictly adhered to WHO guidelines for hookworm detection, focusing solely on the 30-minute timepoint to prevent the clearing of hookworm eggs [38].

In this study, KK2.0 demonstrated significantly higher sensitivity than KK1.0 for identifying *A*. *lumbricoides* at both timepoints. Both methods performed equally well for *T*. *trichiura* at both timepoints and were similar at the 30-minute timepoint for hookworms. These findings indicate that the AI-DP prototype KK2.0 could be cost-effective and has the potential to significantly enhance efforts in STH prevention, owing to its sensitivity and automation.

Measuring accurate STH prevalence is crucial for public health, epidemiology, and effective preventive control programs, facilitating targeted interventions and resource allocation to reduce STH infections and improve public health. In this study, the WHO-recommended KK1.0 method identified *A*. *lumbricoides* (42.9%) as the most common STH, followed by *T*. *trichiuria* (30.1%), and hookworms (2.6%). KK2.0 detected significantly more *A*. *lumbricoides* (59.1%) infections than both TSET and KK1.0. The TSET exhibited a stronger agreement with KK1.0 than with KK2.0. The relatively lower total STH prevalence (<50%) observed in this study was attributed to the MDA program conducted in April 2022 (up to April 30^th^) (**S3 Table**). Overall, the findings of this study hold paramount importance, as decisions regarding public health deworming or MDA campaigns are based on infection intensities, e.g., a decision to treat the population once or twice a year. If the existing reference diagnostic is consistently underperforming, there is a risk of populations being erroneously categorized into a lower intensity range, limiting their access to deworming medications.

Currently, more cost-effective integrated helminth control strategies are replacing individual control programs [39]. These strategies encompass other parasites that can cause infections in addition to STH, including *Enterobius vermicularis* [40], *Hymenolepis nana* [41], *S*. *stercoralis* [42], and *Taenia* [43]. Consequently, the performance of KK1.0, and TSET in detecting intestinal parasites beyond STH was accessed and compared. This evaluation also served as an initial step in training the AI-DP to identify a wider spectrum of intestinal parasites.

With no established standard for egg counts, the results of this study relied on the defined pseudo standard: a sample was deemed truly positive if any diagnostic method reported a positive result at any time during the study. This approach carries the potential for bias, which can be mitigated through the adoption of effective quality control measures, for example, reviewing all stored images to define a ground truth for egg counts. Although AI-DP has demonstrated promising results within a standard laboratory setting, fully unlocking its potential necessitates further investigation and refinement.

### Limitations

There are certain factors that may have impacted the results of this study. The sample size in this study was determined for pragmatic reasons, and as such, it might not have fully captured the diversity or variability present in the broader population.

There are a few methodological limitations that should be considered while interpreting findings from this study. Based on visual analysis of the images obtained with KK2.0 during the initial phase of the study, the lab technicians could not fully understand the timing after homogenization for the KK1.0 and KK2.0 first readings at the 30-minute timepoint. After re-training, the sample preparation and additional experience, images from scans became visibly clearer. Sample preparation is clearly a variable worth considering additional attention in future studies. The hookworm eggs may have hatched during the transportation period of 2–4 hours to the laboratory to prevent clearing of hookworm eggs. However, since samples at the 1-hour timepoint were processed as promptly as feasible, any remaining hookworm eggs should be similarly represented between the two KK methods. Consequently, this occurrence is not anticipated to directly influence the study outcome, specifically the comparison between KK1.0 and KK2.0. Additionally, KK 1.0 was read only once, i.e. a single KK slide instead of both single and duplicate KK readings due to high daily sample flow which posed logistical constraints, especially regarding re-reading samples within the designated time-frame. Considering these challenges, we ensured the reliability of the study findings by implementing a random re-examination of 10% of the KK1.0 results by an independent laboratory technician. This approach helped mitigate potential inconsistencies and upheld the robustness of our findings despite the logistical hurdles encountered. The data collection process was affected by minor technical glitches and user errors in the KK2.0 system. While efforts were made to mitigate these issues, they led to a reduction in the overall data collected. KK2.0 needs improvement based on these experiences. The time taken to read each slide in KK1.0 vs KK2.0 was not recorded, however we anticipated KK2.0 may significantly reduce the time required to obtain results in future mass population studies as not all but only subset of slides would necessitate manual verification.

Three months before the study initiation, a deworming campaign was carried out throughout Peru in May 2022. This was the first campaign since the COVID-19 pandemic disrupted routine deworming efforts. This campaign occurred independent of our study, and we had no influence over its timing in relation to our research initiation. The team strategically calculated the necessary time-frame to conduct the study, aiming to commence closer to the next scheduled deworming in October 2022. The study team tried to optimize timing to ensure some STH positive samples following the deworming campaign. Additionally, it was deemed important to evaluate the KK2.0 technology in a setting that included negative samples, allowing for a comprehensive assessment across a spectrum of infection intensities, from negative to low, moderate, and high. Thus, while the deworming campaign preceding the study might have decreased infection rates and intensity, it also provided an opportunity for our team to investigate potential variations in diagnostic sensitivity of KK2.0 technology, especially concerning low-infection intensity samples following deworming. This circumstance also afforded us a valuable chance to evaluate the performance of the KK2.0 system within a real-world context. Sixth, for KK2.0, the analysis focused solely on AI-identified objects in the field-of-view images. A comprehensive examination of all images by expert users was not conducted. A more thorough review, involving human identification of potential false negatives, is necessary for establishing a reference standard for egg counts. Such an approach is critical for future AI research, where this dataset may serve as a ‘ground truth’, ensuring that all eggs are identified by human experts. In the future, this study’s dataset may be leveraged to train, evaluate, and benchmark the performance of new AI models which may be crucial to further improvement of KK2.0. This step is crucial for advancing the field and ensuring the robustness, accuracy, and reliability of AI algorithms in egg detection and future applications. Furthermore, it is important to extend the comparative analysis between KK1.0 and KK2.0 to other countries and use cases (e.g. as a point-of-care device in remote field settings) and assess the non-inferiority or superiority of KK2.0 over KK1.0 in various settings as per the WHO TPPs for monitoring and evaluation of STH [20], including usability and cost-efficiency.

## Conclusion

In this non-interventional field study for STH, the diagnostic performance of the AI-DP KK2.0 prototype in the identification and quantification of all STH species was comparable to KK1.0, the established standard method capable of meeting the diagnostic requirements for planning, monitoring, and evaluating deworming programs. Notably, the KK2.0 approach exhibited higher sensitivity in detecting *A*. *lumbricoides* at low intensities. This study provides evidence that the KK2.0 prototype can achieve clinical sensitivity and specificity exceeding the desired diagnostic performance outlined in the WHO TPPs (specificity: ≥94%, sensitivity: 60%). Furthermore, the outcomes of this study provide robust evidence for the potential expansion of the AI-DP KK2.0 prototype to detect other common non-STH parasites. Overall, furthering the development of the KK2.0 approach holds promise for developing more accessible and efficient methods for the reliable diagnosis of STH and other parasitic infections. This development paves the way for the development of comprehensive automated digital microscopes aligned with the WHO guidelines for diagnosing neglected tropical diseases.

## Supporting information

S1 Info Study protocol. (PDF)

S2 Info AI egg verification process. (DOCX)

S1 Table Demographics and baseline characteristics. (DOCX)

S2 Table EPG by diagnostic method, STH species, and timepoint—positive samples. (DOCX)

S3 Table Prevalence of STH infections by diagnostics method, STH species and month after MDA. (DOCX)
