# Supplementary material for: Artificial intelligence-based digital pathology for the detection and quantification of soil-transmitted helminths eggs
Source: PLoS Negl Trop Dis. 2024 Sep 30;18(9):e0012492. doi: 10.1371/journal.pntd.0012492 (PMC11488745; doi:10.1371/journal.pntd.0012492)
Supplement: S1 Info — (PDF) [file pntd.0012492.s001.pdf]

Janssen Research and Development*

Non-interventional Study Protocol

To Evaluate a New Innovative Artificial Intelligence Digital Pathology (AI-DP) Tool to Better Detect Soil-transmitted Helminthiasis (STH) in the Context of the VERMOX (Mebendazole) Donation Program for STH

Protocol 108329HEL4001

VERMOX (mebendazole)

* Janssen EMEA is a regional organization that operates through different legal entities in various countries. The legal entity acting as the sponsor for Janssen EMEA studies may vary, such as, but not limited to Janssen Biologics, BV, Janssen-Cilag International NV, or Janssen Pharmaceutica NV. The term "sponsor" is used throughout the protocol to represent these various legal entities. The sponsor is identified on the Contact Information page that accompanies the protocol.

Status: Approved
Date: 26 March 2022
Prepared by: Janssen Research & Development, LLC
EDMS number: EDMS-RIM-645748, 1.0

Compliance: This study will be conducted in compliance with the protocol and applicable regulatory requirements.

Confidentiality Statement
The information provided herein contains Company trade secrets, commercial or financial information that the Company customarily holds close and treats as confidential. The information is being provided under the assurance that the recipient will maintain the confidentiality of the information under applicable statutes, regulations, rules, protective orders or otherwise.

TABLE OF CONTENTS

TABLE OF CONTENTS 2

LIST OF ATTACHMENTS 3

LIST OF IN-TEXT TABLES AND FIGURES 3

PROTOCOL AMENDMENTS 4

SYNOPSIS 5

DATA COLLECTION SCHEDULE 11

LIST OF ABBREVIATIONS AND DEFINITIONS OF TERMS 12

1. INTRODUCTION 13
1.1. Background 13
1.2. Current Treatments 14
1.3. Overall Rationale for the Study 15

2. OBJECTIVES AND HYPOTHESIS 15

3. RESEARCH METHODS 16
3.1. Study Design and Rationale 16
3.1.1. Overview of Study Design 16
3.1.2. Rationale for Study Design 20
3.2. Study Setting 20
3.3. Patient Population 20
3.3.1. Patient Selection Criteria 20
3.3.2. Participating Patient Completion/Withdrawal 21
3.4. Data Sources and Data Collection Methods 22
3.4.1. Soil-transmitted Helminthiasis Therapy 22
3.4.2. Measures of Effectiveness/Clinical Response 22
3.4.2.1. Effectiveness/Clinical Response Measures 22
3.4.2.2. Effectiveness/Clinical Response Parameters 23
3.4.3. Safety Measures 23

4. STATISTICAL ANALYSIS METHODS 23
4.1. Determination of Sample Size 23
4.2. Measure to Minimize Bias 23
4.3. Data Analyses 24
4.3.1. Data Analyses 24
4.3.2. Safety Analyses 26
4.4. Handling of Missing Data 26

5. SAFETY DATA AND COMPLAINT COLLECTION AND REPORTING 26

6. STUDY-SPECIFIC MATERIALS 26

7. ETHICAL AND REGULATORY CONSIDERATIONS 26
7.1. Study-Specific Considerations 26
7.2. Ethics Compliance 27
7.2.1. Independent Ethics Committee or Institutional Review Board 27
7.2.2. Informed Consent 27
7.2.3. Data Protection 28
7.3. Regulatory Documentation 28
7.3.1. Regulatory Approval/Notification 28

7.3.2. Required Prestudy Documentation 28

8. STUDY ADMINISTRATION 29
8.1. Changes to the Protocol 29
8.2. Patient Identification and Enrollment 29
8.3. Source Documentation 30
8.4. Case Report Form Completion 30
8.5. Data Quality Control 31
8.6. Record Retention and Archiving 31
8.7. Study Completion/Termination 31
8.8. Use of Information 32
8.9. Publication Policy 32

9. REFERENCES 34

ATTACHMENTS 35

PARTICIPATING PHYSICIAN AGREEMENT 42

LIST OF ATTACHMENTS

Attachment 1: Sample Processing Form 35
Attachment 2: Demographic Data Collection Form 41

LIST OF IN-TEXT TABLES AND FIGURES

TABLES

Table 1: Fecal Egg Count Thresholds Defining Low, Moderate and Heavy Infection Intensity [2]. The Fecal Egg Counts are Expressed as Eggs per Gram of Stool. 14

FIGURES

Figure 1: Stool Sample Collection and Analysis for Soil-transmitted Helminthiasis in the Jungle Region of Ucayali, Peru 19

PROTOCOL AMENDMENTS

Neither the participating physician nor the sponsor will modify this protocol without a formal amendment. All protocol amendments must be issued by the sponsor, and will be reviewed and approved in accordance with local regulations (see Section 8.1).

There are no amendments to this protocol.

SYNOPSIS

Protocol Title: To Evaluate a New Innovative Artificial Intelligence Digital Pathology (AI-DP) Tool to Better Detect Soil-transmitted Helminthiasis (STH) in the Context of the VERMOX (Mebendazole) Donation Program for STH (v1.0, 26 March 2022)

Sponsor's Responsible Medical Officer: Nancy Cure-Bolt (Main Author)

The term "sponsor" used throughout this document refers to the entities listed in the Contact Information page(s), which will be provided separately.

BACKGROUND AND RATIONALE

Johnson and Johnson (J&J) Global Public Health (GPH), as part of its initiative to improve access to medicines, foster collaborations, and support public health solutions to sustainably advance healthcare worldwide, has, since 2006, donated 2 billion doses of VERMOX (mebendazole) 500 mg tablets to treat intestinal worm infections (also known as soil-transmitted helminthiasis, [STH]) in children living in STH endemic countries as part of a STH control program.

Aligned with the World Health Organization's (WHO) 2030 Neglected Tropical Diseases (NTD) roadmap, J&J GPH is focused on combatting STH infections through donations that aim to decrease STH infection prevalence and reduce STH morbidity. As progress is made against STH, endemic countries can graduate out of donation programs and what little STH burden remains can be incorporated into individual country universal health coverage, where available.

Soil-transmitted helminthiasis control programs are based on a mass drug administration approach – so called preventive chemotherapy (PC) programs to a target population, regardless of individual infection status. The primary microscopic diagnostic technique to support the WHO programs is known as Kato-Katz (KK) thick-smear, in which stool samples are collected, made into a slide smear, and eggs are counted to determine infection intensity. However, limited local diagnostic capacity to assess the prevalence and intensity of infections in a standardized and timely way, is impacting the evaluation and decision-making processes of drug donation utilization within STH country programs. Johnson and Johnson is publicly committed to donating 200 million doses annually of chewable mebendazole through 2025, and to evaluate an accurate and efficient diagnostic tool for detecting STH.

Janssen GPH Research and Development (R&D) has been evaluating an artificial intelligence digital pathology (AI-DP) based on the WHO microscopy diagnostic standard KK thick-smear, in which stool samples are collected, made into a slide smear, inserted into a slide scanner and read to provide an automated egg count without the use of a microscope. An open-source relational database built in MySQL (a database management system) manages the data and allows the custom AI-DP development pipeline.

The AI-DP tool can facilitate drug administration based on prevalence and intensity of infections, as defined by WHO threshold criteria of low, moderate, and heavy intensities.

In Latin America (LATAM), the region's standard of care for monitoring STH prevalence is Tube Spontaneous sEdimentation Technique (TSET, a concentration by sedimentation assay, without centrifugation) which is a qualitative method and differs from the most commonly applied diagnostic method elsewhere, the KK (hence forth will be designated as KK1.0), a quantitative method. The KK1.0 technique is recommended by WHO for helminthiasis diagnosis, however limited reproducibility, a low sensitivity for low intensity infections, and the fact that it needs to be performed on fresh samples means that it is not always the ideal method. This may be particularly true in the context of evaluating large scale interventions aimed at STHs, furthermore, the distance to the central laboratory, combined with limited staff availability has led countries to choose concentration techniques over the KK1.0. Hence, data comparing KK AI-DP (hence forth will be designated as KK2.0) to local approaches is necessary to the development of the tool and to drive future adoption into the LATAM region.

This study is an opportunity to support and accelerate the development and data generation of an innovative diagnostic tool, KK2.0, that could potentially increase the efficiency and impact of mebendazole drug donation to patients around the world.

OBJECTIVES AND HYPOTHESIS

The intent of the study is to evaluate the sensitivity of KK2.0 at detecting STH eggs among school-aged children (SAC) living in selected STH endemic areas of Ucayali, a remote Amazonian region of Peru.

Since a "gold" standard diagnostic test is not available for detection of intestinal parasites, the operational characteristic (sensitivity) will be estimated using the combined results from KK1.0 and KK2.0 at 30-minute and 24-hour timepoints and TSET (within first hour) as a diagnostic pseudo "gold" standard in this study.

**Objectives**	**Endpoints**
**Primary**	
To assess the diagnostic performance of KK2.0 in comparison to KK1.0 at detecting hookworms (*Necator americanus* and *Ancylostoma duodenale*) and *Ascaris lumbricoides* and *Trichuris trichiura* in SAC living in selected areas of Ucayali, a remote Amazonian region of Peru, at the 30-minute and 24-hour timepoints, respectively, of slide preparation.	• Eggs per gram (EPG)<br>• Sensitivity of KK1.0 and KK2.0 at 30-minute and 24-hour timepoints<br>• Agreement in KK1.0 and KK2.0 results at 30-minute and 24-hour timepoints<br>• Percentages of SAC with low, moderate, or heavy intensity of infection by EPG
**Secondary**	
To evaluate the prevalence of STH (hookworms [*Necator americanus* and *Ancylostoma duodenale*], and *Ascaris lumbricoides* and *Trichuris trichiura*) in the selected endemic, remote Amazonian region of Ucayali, Peru	• STH prevalence as a percentage in SAC living in the selected areas of Ucayali, Peru
**Exploratory**	
To assess and compare if KK2.0 with KK1.0 and TSET at identifying intestinal parasites, other than STH.	• Description of intestinal parasites, identified in SAC enrolled in the study

This is a prospective, non-interventional study to evaluate the KK2.0 method and compare its efficiency to KK1.0 at detecting STH. The results will be presented descriptively, the analysis will be exploratory in nature, and no hypotheses are prespecified.

STUDY DESIGN

This is a prospective, non-interventional field study that will be conducted in selected STH endemic areas of Ucayali, a remote Amazonian region of Peru. This trial will evaluate the KK2.0 method and the data collected will aim to advance the goal to strengthen existing STH program surveillance by improving the sensitivity of current testing, and by providing access to timely and higher quality data (more efficient and faster data).

Approximately 2 weeks prior to stool collection, field workers will initially survey the geographical area of Ucayali, Peru, specifically area A, B, C, D, E, and F to identify homes with SAC, aged 5-14 years (Visit 1). The day prior to sample production and collection, field workers will visit the homes of children in the study population, where they will explain the study to parents/guardians and children over the age of 8, obtain informed consent from parents/guardians of SAC aged 5-14 years, and additionally an assent from SAC older than 8 years (Visit 2). Participating households will then receive a sample pot to be collected by the field worker the following day.

Field workers will then collect the stool samples from the consented/assented SAC (Visit 3) and take them to the local laboratory site within 4±1 hours of collecting the samples. At the laboratory, samples will be processed and analyzed for the presence of *Ascaris lumbricoides*, *Trichuris trichiura* and hookworms (*Necator americanus* and *Ancylostoma duodenale;* hence forth will be designated as hookworms) eggs. Results of a STH or other additional parasites identified by TSET (the standard diagnostic method in Peru), will be given to the children's parents/guardians and recommend that they seek treatment.

Within 1 hour of stool sample arriving at the laboratory site, lab technicians will prepare from each sample a TSET assay, and 2 KK thick-smear slides (differentiated as Slide A and Slide B). One technician will prepare the sample for TSET and count the number of *A. lumbricoides*, *T. trichiura*, hookworm eggs, and any other additional identifiable parasites. This will provide qualitative data for later analysis. Two other lab technicians, using the same homogenized stool sample, will prepare the 2 KK thick-smear slides, Slide A and Slide B. Slides A and B will be analyzed within the first 30 minutes (+/- 10 minutes) of slide preparation, in order to count hookworm eggs. Hookworm eggs will clear (ie, eggs will hatch) and disappear from stool samples within this window of time. Slide A will be analyzed via the KK1.0 method that uses manual counts of *A. lumbricoides*, *T. trichiura*, hookworm eggs, and any other identifiable parasites in the 30-minutes timeframe, and Slide B via the KK2.0 method that uses automated egg counts. Slide B will be loaded into the AI-DP slide scanner (aka KK2.0 method) and the system will automatically collect images of the slide. An on-board AI program will process the images, identifying eggs for verification. The slide images and identified eggs will be later uploaded to a cloud database and subsequently uploaded to the study database.

Slide B will be refrigerated overnight to allow the sample to become clearer from sediment and debris so that *A. lumbricoides* and *T. trichiura* eggs can be more easily identified. At the 24±4-hour timepoint, Slide B will undergo a manual (KK1.0) and digital (KK2.0) egg count. Slides will be randomly selected for the KK1.0 method first or KK2.0 method second, or vice versa.

All slides read by the KK1.0 method will be converted to eggs per gram (EPG) of stool statistics and uploaded to the study database. The images of all slides scanned into the AI-DP tool will be uploaded to the EggInspector application in the cloud database which will identify the eggs and the images will be validated by an expert parasitologist in Peru. Validated results will be uploaded to the study database.

Following data collection and upload to the study database, data will undergo quality control, cleaning, and eventually database lock. Ten percent of all slides (ie, KK1.0 and TSET) and 100% of KK2.0 will undergo quality control at each timepoint, where they will be recounted for eggs at the end of the day.

All positively identified specimens for parasites as well as those with negative results by TSET will be summarized at the central laboratory, as per the standard of care in Peru. A signed letterhead by the laboratory's director will be delivered to the children's parents/guardians by the field workers notifying them of their children's infection status (Visit 4) with the recommendation to follow up with their healthcare provider clinic for treatment if positive for any STH or other parasites.

Data collection will be considered complete for a participating child if data collected has been recorded in the electronic case report form (eCRF).

The end of the study will be the last stool sample collected within the study for the last participating child. The overall duration of the study, including recruitment, is expected to be approximately 7 months followed

by a 2-month period for analysis of results. The stool sample collection is expected to be completed within 3 of those 7 months.

STUDY SETTING AND PATIENT POPULATION

There will be no formal study sites in this study. Trained local field workers will visit houses in areas A, B, C, D, E, and F of Ucayali, Peru and will obtain approximately 500 fresh stool sample from consented and assented participating SAC. The study will focus on SAC (age 5–14) since they have a higher burden of STH infection and are a major target of PC programs for treating STH. Samples will be collected and taken to the laboratory for analyses.

DATA SOURCES AND COLLECTION METHODS

The primary data sources for this study will be the Demographic Data Collection Form and the Sample Processing Form. Demographic information of each child will be collected at their home (Visit 2) by the field worker and recorded on the Demographic Data Collection Form. Stool sample analyses will be done at the laboratory and data will be recorded on the Sample Processing Form by the technician. All data will be entered in the eCRF by the lab coordinator, who is an expert lab technician.

Measures of Effectiveness/Clinical Response

At the laboratory, trained laboratory technicians will prepare stool-thick-smear slides for KK1.0, KK2.0, and stool concentrations for the TSET method and will be read manually and by the KK2.0 method at specific timepoints and in accordance with standard process for each test. Slide images read by the AI-DP tool will be uploaded to the EggInspector application in the cloud database, then manually verified by an expert parasitologist in Peru. Validated results will be uploaded to the study database.

Definitions of appropriate measures of effectiveness/clinical response are as follows:

- KK1.0 method: The microscopy diagnostic standard for diagnosing STH infection intensity. Stool samples are made into a slide smear in which eggs are counted.
- KK2.0 method: An improved diagnostic technique over KK1.0 in development that uses AI-DP technology to facilitate egg counting without using a microscope. Using the KK thick smear technique, stool slide smear will be inserted into a slide scanner to provide an automated egg count. At present, this is a hybrid methodology that uses AI-DP and an expert parasitologist verification.
- TSET method: A qualitative method commonly used in LATAM to monitor for STH infection using a concentration by sedimentation assay, without centrifugation.
- Positive to any STH species: If any egg of that STH species is identified by any of the methods.

Measures of Safety

There will be no safety measures in this study as this is a non-interventional study.

STATISTICAL METHODS

Sample Size Determination

The study is designed to generate descriptive data for informative purpose. No formal hypotheses will be tested. The planned sample size of 500 children is based on pragmatic considerations.

Parameters of Interest

The parameter of interest to evaluate diagnostic performance of KK1.0 and KK2.0 will be egg counts and intensity of egg load of low, moderate, or heavy per diagnostic method and per STH species as defined in the Statistical Analysis of Data below.

Statistical Analysis of Data

Descriptive summaries will be provided by diagnostic method and STH species. All continuous variables will be summarized using descriptive statistics, which will include the number of children, mean, standard deviation, median, minimum, and maximum. A 95% confidence interval (CI) will also be provided for EPG and sensitivity. All categorical variables will be summarized using frequencies and percentages. Summary and analysis will be provided by diagnostic method, STH species, and timepoints (30 minutes and 24 hours).

Egg load will be classified into 3 intensity categories as detailed below according to EPG:

- *A. lumbricoides*: Low: <5,000 EPG; Moderate: 5,000-49,999 EPG; Heavy: >50,000 EPG
- *T. trichiura*: Low: <1,000 EPG; Moderate: 1,000-9,999 EPG; Heavy: >10,000 EPG
- Hookworms: Low: <2,000 EPG; Moderate: 2,000-3,999 EPG; Heavy: >4,000 EPG

Since TSET method cannot produce EPG, intensity of each STH species will only be derived for KK1.0 and KK2.0 methods.

Since no "gold" standard for egg counts exist in this study, a diagnostic pseudo "gold" standard will be created. A sample is considered to be truly positive if any diagnostic method comes back with a positive reading at any time of the study.

Number of total positive samples for a STH species is defined as

$N(+) = N(KK1.0(+)_{30m} \cup KK2.0(+)_{30m} \cup KK1.0(+)_{24h} \cup KK2.0(+)_{24h} \cup TSET(+))$

Where $KK1.0(+)_{30m}$ indicates a positive sample based on KK1.0 at 30 minutes, $KK2.0(+)_{30m}$ indicates a positive sample based on KK2.0 at 30 minutes, $KK1.0(+)_{24h}$ indicates a positive sample based on KK1.0 at 24 hours, $KK2.0(+)_{24h}$ indicates a positive sample based on KK2.0 at 24 hours, and TSET(+) indicates a positive sample based on TSET which is performed only once during the study.

Analysis of primary endpoints

Summary of EPG will be provided by diagnostic method, STH species, and timepoint. A permutation test will be employed for comparison of EPG between different diagnosis methods.

Sensitivity for KK1.0 at 30 minutes will be calculated as

$$= \frac{N(KK(1.0(+)_{30m})}{N(+)}$$

Where $N(KK1.0(+)_{30m})$ is the number of positive samples based on KK1.0 method at 30 minutes. Sensitivity for other diagnostic methods and other timepoints will be calculated similarly.

A permutation test will be employed for comparison of sensitivity between different diagnosis methods.

Summary of percentages of SAC with low, moderate, or heavy infection intensity will be provided.

2x2 (eggs, no eggs) and 3x3 (low, moderate, or heavy infection intensity) tables based on egg load will be provided to evaluate agreement between different test methods. Weighted Kappa statistics will be provided.

Analysis of secondary endpoints

Prevalence of STH infection is defined as the percentage of children with positive egg count readings. Summary of prevalence will be provided by diagnostics method and STH species. If a child is diagnosed as positive for a STH species during anytime of the study, this child is considered STH infected.

Summary of the qualitative readings (1+, 2+, 3+) from TSET will also be provided.

Analysis of exploratory endpoints

Descriptive summary of intestinal parasites identified in SAC enrolled in the study will be provided by diagnostic method (KK2.0, KK1.0, and TSET), timepoints (30 minutes and 24 hours), and intestinal parasite species.

DATA COLLECTION SCHEDULE

Data Collection	**Visit 1** (2 weeks prior to samole collection)	**Visit 2** (1 day prior to sample collection)	**Visit 3** (Stool collection)	**Visit 4** (Results Notification)
**Study Procedure**				
**Sc1·eenin2/Administrative**				
Households with SAC identified bv FW	X			
FW provides general overview of studv to households	X			
FW explains study to children and parents/guardians		X		
Infonned consent fonn/assent GCF)•		X		
Demographics		X		
Inclusion/exclusion criteria[b]		X		
**Clinical Laboratorv Tests**				
Stool collection			X	
Slide analvsis begins			X	
Stool sample results are delivered to parents/guardians and recommendations for treatment to be orovided[0]				X

Abbreviations: FW=field workers, SAC=school-aged children

a. Must be signed before first study-related activity.

b. Minimum criteria for the availability of documentation supporting the eligibility criteria will be described in the full protocol.

c. Treatment recommendations are based of standard of care in the region.

LIST OF ABBREVIATIONS AND DEFINITIONS OF TERMS

Abbreviations

AI	artificial intelligence
AI-DP	artificial intelligence digital pathology
CI	confidence interval
eCRF	electronic case report form
eDC	electronic data capture
EPG	eggs per gram
FOIA	Freedom of Information Act
GPH	Global Public Health
ICF	informed/assent consent form
IEC/IRB	Independent Ethics Committee/Institutional Review Board
J&J	Johnson and Johnson
KK	Kato-Katz
KK1.0	Kato-Katz 1.0
KK2.0	KK AI-DP
LATAM	Latin America
NTD	Neglected Tropical Diseases
PC	preventive chemotherapy
R&D	Research and Development
STH	soil-transmitted helminthiasis
TSET	Tube Spontaneous sEdimentation Technique
WHO	World Health Organization

Definition of Terms

Study	The term "study" indicates a non-interventional clinical activity where patient level data may be systematically collected and/or analyzed for medically important results/outcomes, for research purposes. The use of this term in no way implies that any interventional treatments or procedures, planned or otherwise, have been provided or performed.
Prospective study	A study in which the outcome of interest occurs after the research begins.
School-aged children	School-aged children that are most susceptible to soil-transmitted helminthiasis.

1. INTRODUCTION

1.1. Background

Johnson and Johnson (J&J) Global Public Health (GPH) aims to improve access to medicines, foster collaborations, and support public health solutions to sustainably advance healthcare worldwide. Since 2006, J&J has cumulatively donated 2 billion doses of VERMOX (mebendazole) 500 mg tablets to treat intestinal worm infections (also known as soil-transmitted helminthiasis, [STHs]) in children living in STH endemic countries.

With goals of supporting the elimination of STH morbidity as a public health problem, J&J is publicly committed to an annual donation of 200 million doses of chewable mebendazole through 2025. Aligned with the World Health Organization's (WHO) 2030 Neglected Tropical Diseases (NTD) roadmap, J&J GPH is focused on combatting STH infections through donations that aim to decrease STH infection prevalence and reduce STH morbidity. As progress is made against STH, endemic countries can graduate out of donation programs and what little STH burden remains can be incorporated into individual country universal health coverage, where available.

Unification of different NTD stakeholders together, can significantly contribute to the control and elimination of these NTDs. Soil-transmitted helminthiasis control programs are based on a mass drug administration to a target population, regardless of individual infection status – so called preventive chemotherapy (PC) programs. The primary microscopic diagnostic technique to support the WHO programs is known as Kato-Katz (KK) thick-smear, in which stool samples are collected, made into a slide smear, and eggs are counted to determine infection intensity. However, limited local diagnostic capacity to assess the prevalence and intensity of infections in a standardized and timely way, is impacting the evaluation and decision-making processes of drug donation utilization within STH country programs.

This study is an opportunity to support and accelerate the development and data generation of an innovative diagnostic tool that could potentially increase the efficiency and impact of mebendazole drug donation to patients around the world.

Janssen GPH Research and Development (R&D) has been evaluating an artificial intelligence digital pathology (AI-DP) based on the KK thick-smear, to achieve WHO goal for the Diagnostic Target Profile of the STH as part of J&J contribution to 2030 NTD goals. The prototype unit has travelled for demonstration and field testing to Tanzania, Cambodia, Kenya, Ethiopia, Washington DC (COR-NTD Innovation Lab) and Belgium. During the course of evaluation, 7,780 field of view images (of approximately 800 µm x 600 µm) were manually reviewed to verify image quality, image focus, locations and classes of STH eggs. A total of 16,990 parasite eggs have been marked-up and form the ground truth data for training artificial intelligence (AI) models. An open- source relational database built in MySQL (a database management system) manages the data and allows the custom AI-DP development pipeline.

The performance of the technology is summarized as follows:

- *Ascaris lumbricoides*: Sensitivity 96%, Specificity 98%, Precision 98%, Accuracy 97%
- *Trichuris trichiura*: Sensitivity 97%, Specificity 98%; Precision 93%, Accuracy 98%
- Hookworms (*Necator americanus* and *Ancylostoma duodenale*): Sensitivity 96%, Specificity 99%; Precision 97%, Accuracy 99% [1]

Since the frequency (ie, annually or bi-annually) of drug administration in PC programs are based on prevalence and intensity of infections, the AI-DP tool is a more sensitive diagnostic method and that can lead to more successful implementation of STH programs. The WHO has defined threshold criteria to describe low, moderate and heavy infection intensity and is presented below in Table 1 [2].

Table 1: Fecal Egg Count Thresholds Defining Low, Moderate and Heavy Infection Intensity [2]. The Fecal Egg Counts are Expressed as Eggs per Gram of Stool.

	Low	Moderate	Heavy
Ascaris lumbricoides	1–4,999	5,000–49,999	>50,000
Trichuris trichiura	1–999	1,000–9,999	>10,000
Hookworms	1–1,999	2,000–3,999	>4,000

The current AI-DP prototype is now ready to be piloted in Latin America (LATAM). Soil-transmitted helminthiasis infect a significant proportion of Latin Americans, particularly in poor neighborhoods and native communities. It is estimated that in LATAM and the Caribbean at least 13.9 million preschool-aged (pre-SAC) and 35.4 million school-aged children (SAC) are at risk of infections by STH [3]. These infections rarely cause death, but their public health consequences are manifest in the chronic, insidious effects that the condition has such as malnutrition, anemia, impeded growth and increased susceptibility to other infections. In both pre- SAC (1-4 years) and SAC (5-14 years), the age-groups most at risk, STH infection stunts physical growth and impairs the development of cognitive function, hampering performance and attendance within the education system and ultimately hindering economic development [4-6]. The recommended control strategy is a population-based approach whereby all members of a target population receive treatment with anthelminthic drugs, usually albendazole or mebendazole, regardless of infection status. This kind of PC known as mass drug administration, is relatively inexpensive and justified as the risks for uninfected individuals receiving the drug is low [7]. However, a diagnostic tool such as the AI-DP can more accurately and efficiently estimate the prevalence of STH infection in LATAM, thus making drugs more readily available to this population through a more targeted donation program.

1.2. Current Treatments

Soil-transmitted helminths (STH) include *A. lumbricoides*, *T. trichiura*, and 2 hookworm species, namely *Necator americanus* and *Ancylostoma duodenale* (hence forth will be designated as hookworms). Global estimates indicate that more than 1.5 billion people are infected with at least one of the 4 STH species [8]. They are responsible for an estimated 3.3 million disability- adjusted life years of which 1.2 million occurred in children which is the highest burden among all

NTDs [9]. Preventive chemotherapy is the main strategy to control the morbidity caused by STHs. This entails the periodic administration of a single, oral dose of albendazole (ALB; 400 mg) or mebendazole (MEB; 500 mg) mainly to children through school-based PC program [10].

1.3. Overall Rationale for the Study

The validation of the AI-DP tool in LATAM is critical as the region's standard of care for monitoring STH prevalence is Tube Spontaneous sEdimentation Technique (TSET, a concentration by sedimentation assay, without centrifugation) which is a qualitative method and differs from the most commonly applied diagnostic method elsewhere, the KK (hence forth will be designated as KK1.0), a quantitative method. The KK1.0 technique is recommended by WHO for helminthiasis diagnosis [5], however limited reproducibility, a low sensitivity for low intensity infections, and the fact that it needs to be performed on fresh samples means that it is not always the ideal method. This may be particularly true in the context of evaluating large scale interventions aimed at STHs, furthermore, the distance to the central laboratory, combined with limited staff availability has led countries to choose concentration techniques over the KK1.0. [11]. Hence, data comparing KK AI-DP (hence forth will be designated as KK2.0) to local approaches is necessary to the evaluation of the tool and to drive future adoption into the LATAM region.

Counting of helminth eggs requires a minimum amount of laboratory equipment and experienced laboratory technicians to ensure quality of the data obtained; and it is essential that samples are analyzed quickly after collection to ensure visualization of hookworm eggs. This has prompted the search for an improved diagnostic method, the KK2.0 technique which uses AI-DP to facilitate egg counts without using a microscope, uses automated egg counting, and submission of results from remote locations, even if no internet connection is available at the data collection location.

2. OBJECTIVES AND HYPOTHESIS

Research Question

The intent of the study is to evaluate the sensitivity of KK2.0 at detecting STH eggs among SAC living in selected STH endemic areas of Ucayali, a remote Amazonian region of Peru.

Since a "gold" standard diagnostic test is not available for detection of intestinal parasites, the operational characteristic (sensitivity) will be estimated using the combined results from KK1.0 and KK2.0 at 30-minute and 24-hour timepoints and TSET (within first hour) (see Objectives below and Figure 1 for schematic flow chart) as a diagnostic pseudo "gold" standard in this study [12].

Objectives

**Objectives**	**Endpoints**
**Primary**	
To assess the diagnostic performance of KK2.0 in comparison to KK1.0 at detecting hookworms (*Necator americanus* and *Ancylostoma duodenale*) and *Ascaris lumbricoides* and *Trichuris trichiura* in SAC living in selected areas of Ucayali, a remote Amazonian region of Peru, at the 30-minute and 24-hour timepoints, respectively, of slide preparation.	• Eggs per gram (EPG)<br>• Sensitivity of KK1.0 and KK2.0 at 30-minute and 24-hour timepoints<br>• Agreement in KK1.0 and KK2.0 results at 30-minute and 24-hour timepoints<br>• Percentages of SAC with low, moderate, or heavy intensity of infection by EPG
**Secondary**	
To evaluate the prevalence STH (hookworms [*Necator americanus* and *Ancylostoma duodenale*], and *Ascaris lumbricoides* and *Trichuris trichiura*) in the selected endemic, remote Amazonian region of Ucayali, Peru.	• STH prevalence as a percentage in SAC living in the selected areas of Ucayali, Peru
**Exploratory**	
To assess and compare if KK2.0 with KK1.0 and TSET at identifying intestinal parasites, other than STH	• Description of intestinal parasites, identified in SAC enrolled in the study

Hypothesis

This is a prospective, non-interventional study to evaluate the KK2.0 method and compare its efficiency to KK1.0 at detecting STH. The results will be presented descriptively, the analysis will be exploratory in nature, and no hypotheses are prespecified.

3. RESEARCH METHODS

3.1. Study Design and Rationale

3.1.1. Overview of Study Design

This is a prospective, non-interventional field study that will be conducted in selected STH endemic areas of Ucayali, a remote Amazonian region of Peru. This trial will evaluate the KK2.0 method and the data collected will aim to advance the goal to strengthen existing STH program surveillance by improving the sensitivity of current testing, and by providing access to timely and higher quality data (more efficient and faster data).

Approximately 2 weeks prior to stool collection, field workers will initially survey the geographical area of Ucayali, Peru, specifically area A, B, C, D, E, and F (refer to map of Ucayali, Peru), to identify homes with SAC, aged 5-14 years (Visit 1). The day prior to sample production and collection, field workers will visit the homes of children in the study population, where they will explain the study to parents/guardians and children over the age of 8, obtain informed consent

from parents/guardians of SAC aged 5-14 years, and additionally an assent from SAC older than 8 years (Visit 2). Participating households will then receive a sample pot to be collected by the field worker the following day.

Field workers will then collect the stool samples from the consented/assented SAC (Visit 3) and take them to the local laboratory site within 4±1 hours of collecting the samples. At the laboratory, samples will be processed and analyzed for the presence of *A. lumbricoides*, *T. trichiura*, and hookworm eggs. Results of a STH or other additional parasites identified by TSET (the standard diagnostic method in Peru), will be given to the children's parents/guardians and recommended that they seek treatment.

Within 1 hour of stool sample arriving at the laboratory site, lab technicians will prepare from each sample a TSET assay, and 2 KK thick-smear slides (differentiated as Slide A and Slide B; see Figure 1 for schematic flow chart and Peru KK2.0 Laboratory Protocol for a narrative description). One technician will prepare the sample for TSET and count the number of *A. lumbricoides, T. trichiura*, hookworm eggs, and any other additional identifiable parasites. This will provide qualitative data for later analysis. Two other lab technicians, using the same homogenized stool sample, will prepare the 2 KK thick-smear slides, Slide A and Slide B. Slides A and B will be analyzed within the first 30 minutes (+/- 10 minutes) of slide preparation, in order to count hookworm eggs. Hookworm eggs will clear (ie, eggs will hatch) and disappear from stool samples within this window of time. Slide A will be analyzed via the KK1.0 method that uses manual counts of *A. lumbricoides, T. trichiura,* hookworm eggs, and any other identifiable parasites in the 30-minutes timeframe, and Slide B via the KK2.0 method that uses automated egg counts. Slide B will be loaded into the AI-DP slide scanner (aka KK2.0 method) and the system will automatically collect images of the slide. An on-board AI program will process the images, identifying eggs for verification. The slide images and identified eggs will be later uploaded to a cloud database and subsequently uploaded to the study database. See KK2.0 AID2STOP User Manual.

Slide B will be refrigerated overnight to allow the sample to become clearer from sediment and debris so that *A. lumbricoides* and *T. trichiura* eggs can be more easily identified. At the 24±4-hour timepoint, Slide B will undergo a manual (KK1.0) and digital (KK2.0) egg count. Slides will be randomly selected for the KK1.0 method first or KK2.0 method second, or vice versa.

All slides read by the KK1.0 method will be converted to eggs per gram (EPG) of stool statistics and uploaded to the study database. The images of all slides scanned into the AI-DP tool will be uploaded to the EggInspector application (see EggInspector Quick Start Guide) in the cloud database which will identify the eggs and the images will be validated by an expert parasitologist in Peru. Validated results will be uploaded to the study database.

Following data collection and upload to the study database, data will undergo quality control, cleaning, and eventually database lock. Ten percent of all slides (ie, KK1.0 and TSET) and 100% of KK2.0 will undergo quality control at each timepoint, where they will be recounted for eggs at the end of the day.

All positively identified specimens for parasites as well as those with negative results by TSET will be summarized at the central laboratory, as per the standard of care in Peru. A signed letterhead by the laboratory’s director will be delivered to the children’s parents/guardians by the field workers notifying them of their children’s infection status (Visit 4) with the recommendation to follow up with their healthcare provider clinic for treatment if positive for any STH or other parasites.

The collection of stool samples will be conducted for approximately 3 months followed by a 2-month period for analysis of results.

The different steps of the trials are schematized in Figure 1.

Figure 1: Stool Sample Collection and Analysis for Soil-transmitted Helminthiasis in the Jungle Region of Ucayali, Peru

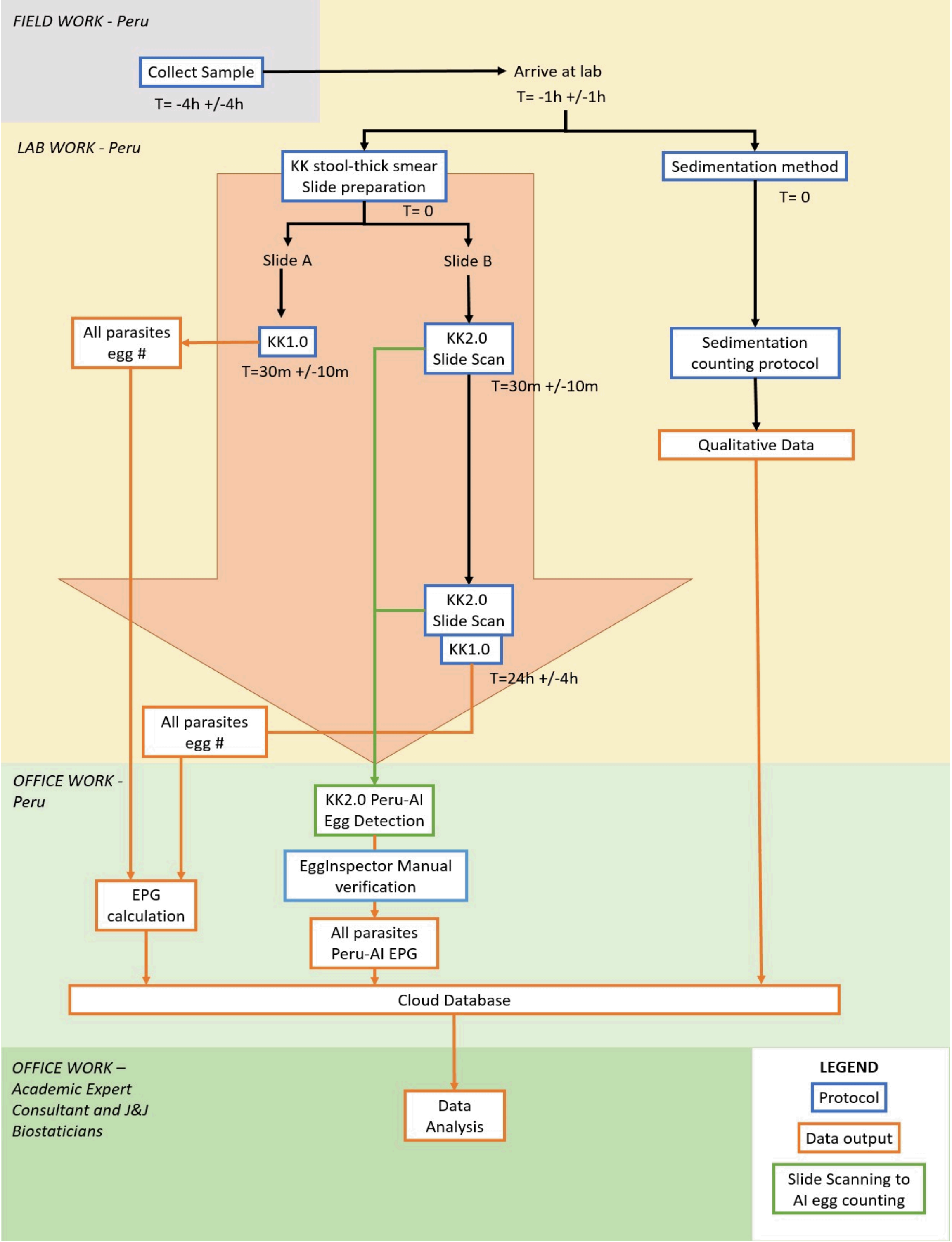

Abbreviations: AI=artificial intelligence, EPG=eggs per gram, KK=Kato Katz

Further details are provided in Section 3.2 and Section 3.3, respectively.

Prior to data collection, all children and/or their legally acceptable representative where applicable must sign a participation informed/assent consent form (ICF) allowing data collection and source data verification in accordance with local requirements and/or sponsor policy.

The end of the study will be the last stool sample collected within the study for the last participating child. The overall duration of the study, including recruitment, is expected to be approximately 7 months followed by a 2-month period for analysis of results. The stool sample collection is expected to be completed within 3 of those 7 months.

The expected frequency and timing of data collection in this non-interventional study are summarized in the DATA COLLECTION SCHEDULE presented after the synopsis.

3.1.2. Rationale for Study Design

This prospective, non-interventional field study design is an effective way of collecting data in STH endemic regions to evaluate an improved STH diagnostic tool (AI-DP) over the standard approaches to allow for a more accurate and efficient data collection, with the aim of promoting a more targeted use of STH drug donations. The incorporation of this tool into STH monitoring programs in STH endemic countries, such as in the Ucayali region of Peru, will help to overcome the diagnostic issues of accessibility, affordability and quality, thus ensuring that data reporting is fully integrated into national health data systems for more efficient and precise targeted drug donations.

3.2. Study Setting

There will be no formal study sites in this study. Trained local field workers will visit houses in areas A, B, C, D, E, and F as indicated in the map of Ucayali, Peru and will obtain approximately 500 fresh stool sample from consented and assented participating SAC. Samples will be collected and taken to the laboratory for analyses.

3.3. Patient Population

School-aged children in selected STH endemic areas of Ucayali, a remote Amazonian region of Peru is the target patient population in this study. The study will focus on SAC (age 5–14) since they have a higher burden of STH infection and are a major target of PC programs for treating STH. An informed consent will be obtained from parents of SAC, aged 5-14 years, and an additional assent will be obtained from SAC older than 8 years. All SAC in a household encounter by the field worker will be asked to provide a fresh stool sample.

3.3.1. Patient Selection Criteria

All children who meet the selection criteria below will be encouraged to participate by the field workers.

Each field worker will assess children to determine their eligibility for sample collection within the study based on the selection criteria. If there is a question about any of the selection criteria,

the field worker should consult with the appropriate sponsor representative before enrolling the child.

Inclusion Criteria

Each potential child participant must satisfy the following criteria to be eligible for data collection in this study:

1. Male or female aged 5 to 14 years of age, inclusive, as this group is at high risk for STH morbidity [13]

2. Child is otherwise in healthy condition as stated by their parents

3. Parent(s)/guardian(s) of children must sign, and/or their legally acceptable representative where applicable must sign, a ICF indicating that they understand the purpose and procedures required for the study and that they are willing to have their child participate in the study

4. Child of 8 years or older has signed an assent document indicating that they understand the purpose of the study and procedures required for the study, and are willing to participate in the study

5. Child is able to provide an adequate stool sample as specified on stool collection container

6. Parent/Guardian and child speak Spanish

Exclusion Criteria

Potential child participant who meets any of the following criteria will not be eligible for this study:

1. Child has active diarrhea (defined as the passage of 3 or more loose or liquid stools per day) at baseline

2. Child has an acute medical condition or is experiencing a severe concurrent medical condition

3. Child has received anthelmintic treatment within 90 days prior to study entry

3.3.2. Participating Patient Completion/Withdrawal

Data collection will be considered complete for a participating child if data collected has been recorded in the electronic case report form (eCRF).

A child participant will be withdrawn from data collection within the study for any of the following reasons:

- Withdrawal of consent
- Decision by the sponsor during the study (eg, due to major protocol violation, including failure to meet the study selection criteria)
- Liquid diarrhea on the day of sample collection

3.4. Data Sources and Data Collection Methods

The DATA COLLECTION SCHEDULE that follows the synopsis summarizes the expected frequency and timing of children contact in this non-interventional study, which must be in accordance with clinical practice, and subsequent data collection.

The primary data sources for this study will be the Demographic Data Collection Form (Attachment 2) and the Sample Processing Form (Attachment 1). Demographic information of each child will be collected at their home (Visit 2) by the field worker and recorded on the Demographic Data Collection Form. Stool sample analyses will be done at the laboratory and data will be recorded on the Sample Processing Form by the lab technician. All data will be entered in the eCRF by the lab coordinator, who is an expert lab technician.

Where available, the following items are to be documented at baseline:

- Demographic data
- Collection of stool sample
- Measures of effectiveness/clinical response (see Section 3.4.2)

3.4.1. Soil-transmitted Helminthiasis Therapy

This study will consist of a stool specimen collection and baseline data collection and no therapy will be investigated.

3.4.2. Measures of Effectiveness/Clinical Response

3.4.2.1. Effectiveness/Clinical Response Measures

At the laboratory, trained laboratory technicians will prepare stool-thick-smear slides for KK1.0, KK2.0, and stool concentrations for the TSET method and will be read manually and by the KK2.0 method at specific timepoints and in accordance with standard process for each test. Slide images read by the AI-DP tool will be uploaded to the EggInspector application in the cloud database (see EggInspector Quick Start Guide), then manually verified by an expert parasitologist in Peru. Validated results will be uploaded to the study database.

Definitions of appropriate measures of effectiveness/clinical response are as follows:

- KK1.0 method: The microscopy diagnostic standard for diagnosing STH infection intensity. Stool samples are made into a slide smear in which eggs are counted.
- KK2.0 method: An improved diagnostic technique over KK1.0 in development that uses AI-DP technology to facilitate egg counting without using a microscope. Using the KK thick

smear technique, stool slide smear will be inserted into a slide scanner to provide an automated egg count. At present, this is a hybrid methodology that uses AI-DP and an expert parasitologist verification.

- TSET method: A qualitative method commonly used in LATAM to monitor for STH infection using a concentration by sedimentation assay, without centrifugation.
- Positive to any STH result: Any sample with egg(s) of any STH species identified by any of the diagnostic methods.

3.4.2.2. Effectiveness/Clinical Response Parameters

Parameter of Interest

The parameter of interest to evaluate diagnostic performance of KK1.0 and KK2.0 will be egg counts and intensity of egg load of low, moderate, or heavy per diagnostic method and per STH species as defined in the Data Analyses in Section 4.3 below.

3.4.3. Safety Measures

There will be no safety measures in this study as no Janssen marketed medicinal product is under study in this non-interventional study.

4. STATISTICAL ANALYSIS METHODS

Statistical analyses will be performed by or under the authority of the sponsor. A general description of the planned statistical methods to be used to analyze the data collected in this study is presented in the following subsections.

4.1. Determination of Sample Size

The study is designed to generate descriptive data for informative purpose. No formal hypotheses will be tested. The planned sample size of 500 children is based on pragmatic considerations.

The sample size will be based on enrolling SAC in the in selected STH endemic areas of Ucayali, a remote Amazonian region of Peru. The sample size is considered adequate to evaluate an improved diagnostic method (KK2.0) over the standard of testing for monitoring STH prevalence in LATAM.

4.2. Measure to Minimize Bias

To reduce potential bias during data collection and evaluation, at the 24-hour timepoint slides will be randomly selected for the manual counting (KK1.0 method) first or the KK2.0 slide scanning method second, or vice versa. Randomization will be conducted via sealed envelopes containing a code assigned to the method, ie, KK1.0 or KK2.0, that the lab technician will use first when reading Slide B. Each day the technicians will have 12 Slide Bs to read. They will select an envelope and follow the order of which method to use first and second when reading Slide B.

4.3. Data Analyses

4.3.1. Data Analyses

Descriptive summaries will be provided by diagnostic method and STH species. All continuous variables will be summarized using descriptive statistics, which will include the number of children, mean, standard deviation, median, minimum, and maximum. A 95% confidence interval (CI) will also be provided for EPG and sensitivity. All categorical variables will be summarized using frequencies and percentages. Summary and analysis will be provided by diagnostic method, STH species, and timepoints (30 minutes and 24 hours). It should be noted that at the 24±4-hour timepoint the same slide (Slide B), will be evaluated by both the KK1.0 and KK2.0 methods. While at the 30-minute timepoint, different slides (slides A and B) from the same stool sample will be evaluated by the same 2 methods. Therefore, extra caution should be exercised when comparing results from the different methods at the 30-minute timepoint since 2 different slides are being analyzed.

Egg load will be classified into 3 intensity categories as detailed below according to EPG:

- *A. lumbricoides*: Low: <5,000 EPG; Moderate: 5,000-49,999 EPG; Heavy: >50,000 EPG
- *T. trichiura*: Low: <1,000 EPG; Moderate: 1,000-9,999 EPG; Heavy: >10,000 EPG
- Hookworms: Low: <2,000 EPG; Moderate: 2,000-3,999 EPG; Heavy: >4,000 EPG

Since TSET method cannot produce EPG, intensity of each STH species will only be derived for KK1.0 and KK2.0 methods.

Since no “gold” standard for egg counts exist in this study, a diagnostic pseudo “gold” standard will be created. A sample is considered to be truly positive if any diagnostic method comes back with a positive reading at any time of the study.

Number of total positive samples for a STH species is defined as

$$N(+) = N(KK1.0(+)_{30m} \cup KK2.0(+)_{30m} \cup KK1.0(+)_{24h} \cup KK2.0(+)_{24h} \cup TSET(+))$$

Where $KK1.0(+)_{30m}$ indicates a positive sample based on KK1.0 at 30 minutes, $KK2.0(+)_{30m}$ indicates a positive sample based on KK2.0 at 30 minutes, $KK1.0(+)_{24h}$ indicates a positive sample based on KK1.0 at 24 hours, $KK2.0(+)_{24h}$ indicates a positive sample based on KK2.0 at 24 hours, and TSET(+) indicates a positive sample based on TSET which is performed only once during the study.

Analysis of Primary Endpoints

Summary of EPG will be provided by diagnostic method, STH species, and timepoint. A permutation test (5,000 iterations) will be employed for comparison of EPG between different diagnosis methods (KK1.0 and KK2.). The 95% CI will be determined based on bootstrap analysis (5,000 iterations). To preserve the correlation of egg count readings by different diagnostic methods from either different samples or same smear of the same participant, permutations will be done within each participant, not across all participants (ie, in a permutation, once an egg count

reading is randomly assigned to one of the 2 diagnostic methods, the other reading from the same participant will be automatically assigned to the other diagnostic method). Re-sampling in bootstrap will be performed at participant level, and the egg count reading will remain with the same diagnostic method as performed.

Sensitivity for KK1.0 at 30 minutes will be calculated as

$$= \frac{\mathrm{N(KK(1.0(+)_{30m})}}{\mathrm{N(+)}}$$

Where $\mathrm{N(KK1.0(+)_{30m})}$ is the number of positive samples based on KK1.0 method at 30 minutes. Sensitivity for other diagnostic methods and other timepoints will be calculated similarly.

A permutation test (5,000 iterations) will be employed for comparison of sensitivity between different diagnosis methods and the 95% CI will be determined based on bootstrap analysis (5,000 iterations) in similar approach as for EPG. Sensitivity will be calculated for each of the 5,000 iterations, and p-value and 95% CI will be constructed.

Summary of percentages of SAC with low, moderate, or heavy infection intensity will be provided.

2x2 (eggs, no eggs) and 3x3 (low, moderate, or heavy infection intensity) tables based on egg load will be provided to evaluate agreement between different test methods. Weighted Kappa statistics will be provided.

Analysis of Secondary Endpoints

Prevalence of STH infection is defined as the percentage of children with positive egg count readings. Summary of prevalence will be provided by diagnostics method and STH species. If a child is diagnosed as positive for a STH species during anytime of the study, this child is considered STH infected.

Summary of the qualitative readings (1+, 2+, 3+) from TSET will also be provided.

Analysis of Exploratory Endpoints

Descriptive summary of intestinal parasites identified in SAC enrolled in the study will be provided by diagnostic method (KK2.0, KK1.0, and TSET), timepoints (30 minutes and 24 hours), and intestinal parasite species.

4.3.2. Safety Analyses

There will be no safety measures in this study as this is a non-interventional study.

4.4. Handling of Missing Data

Only data available will be used for the primary analysis. Statistical and analytic procedures can be applied if deemed appropriate to the handling of missing data for sensitivity analysis.

5. SAFETY DATA AND COMPLAINT COLLECTION AND REPORTING

Since no Janssen marketed medicinal product is under study in this non-interventional study, no adverse events will be collected.

6. STUDY-SPECIFIC MATERIALS

The field workers and laboratory technicians will be provided with the following study-specific materials, and in accordance with their respective role, where applicable:

- Artificial Intelligence- Digital Pathology Tool Protocol
- Informed Consent Form and assent consent form
- Measurement instruments/Study supportive guidelines/User manuals
- EggInspector Quick Start Guide
- Laboratory and KK2.0 technical protocol (KK2.0 AID2STOP User Manual)
- Electronic Data Capture (eDC) Manual
- Personal Protective Equipment
- Sample Processing Form (see Attachment 1)
- Demographic Data Collection Form (see Attachment 2)
- Ucayali, Peru sectional map (A, B, C, D, E, F, and 0)

7. ETHICAL AND REGULATORY CONSIDERATIONS

7.1. Study-Specific Considerations

Prior to data collection, potential participating children (and/or a legally acceptable representative where applicable) will be fully informed of the observational nature of the study, in that the sponsor intends to collect information and follow the course of therapy in clinical practice. They will be told that their consent to allow collection of information within the context of this non-interventional study is voluntary and may be withdrawn at any time. Only children of parent(s) who are fully able to understand the nature of the study and provide their consent, and assent by child of 8 years or older, voluntarily will be enrolled.

When referring to the signing of the ICF, the terms legal guardian and legally acceptable representative refer to the legally appointed guardian of the child with authority to authorize participation in research. For each child, his/her parent(s) (preferably both parents, if available) or a

legally acceptable representative(s), as required by local regulations, must give written consent (permission) according to local requirements after the nature of the study has been fully explained and before any data collection. Assent must be obtained from children (minors) capable of understanding the nature of the study, typically children aged 8 years or older, depending on the institutional policies. For the purposes of this study, all references to children who have provided consent (and assent as applicable) refers to the children and his/her parent(s) or the child's legal guardian(s) or legally acceptable representative(s) who have provided consent according to this process. Minors who assent to a study and later withdraw that assent should not be maintained in the study against their will, even if their parents still want them to participate.

7.2. Ethics Compliance

7.2.1. Independent Ethics Committee or Institutional Review Board

The Regional Ethical Committee will review all documents. This committee is part of the National Institute of Health, which is an organ of the Ministry of Health.

7.2.2. Informed Consent

Each parent/guardian must sign an ICF, and an assent by children of 8 years or older, allowing data collection and source data verification in accordance with local requirements and sponsor policy. The ICF and assent must be signed before collection of any child’s data.

The ICF and assent form that is/are used must be reviewed and approved in accordance with local regulations, applicable regulatory requirements and sponsor policy, and must be in a language that the child can read and understand.

Before enrollment in the study, the field worker must explain to the children and/or their legally acceptable representatives their involvement in the study and data protection. Children will be informed that their participation in the study is voluntary and that they may withdraw consent for data collection at any time. They will be informed that choosing not to participate in this study will not affect the standard of care and access to healthcare the child may need.

The child or legally acceptable representative will be given sufficient time to read the ICF and assent and will be given the opportunity to ask questions. After this explanation and before entry into the study, consent and assent should be appropriately recorded by means of either the child's and/or his/her legally acceptable representative's personally dated signature. After having obtained the consent and assent, a copy of the ICF and assent must be provided to the child and parents/guardians.

If the child and/or legally acceptable representative is unable to read or write, an impartial witness should be present for the entire informed consent process (which includes reading and explaining all written information) and should personally date and sign the ICF and assent after the oral consent of the child or legally acceptable representative is obtained.

Children (minors) or patients who are unable to comprehend the information provided can be enrolled only after obtaining consent of a legally acceptable representative. Assent must be

obtained from children (minors) capable of understanding the nature of the study, ie, children 8 years of age and older, depending on the institutional policies. Written assent should be obtained from children who are able to write. A separate assent form written in language the child can understand should be developed for adolescents. After having obtained the assent, a copy of the assent form must be given to the child, and to the child's parent and/or legally acceptable representative.

7.2.3. Data Protection

Privacy of Personal Data

The collection and processing of personal data from children enrolled in this study will be limited to those data that are necessary to fulfill the objectives of the study.

These data must be collected and processed with adequate precautions to ensure confidentiality and compliance with applicable data privacy protection laws and regulations. Appropriate technical and organizational measures to protect the personal data against unauthorized disclosures or access, accidental or unlawful destruction, or accidental loss or alteration must be put in place. Sponsor personnel whose responsibilities require access to personal data agree to keep the identity of participating children confidential.

The ICF and assent obtained from the child (or his/her legally acceptable representative) includes explicit consent for the processing of personal data and for study-related data monitoring and Independent Ethics Committee/Institutional Review Board (IEC/IRB) review. This consent also addresses the transfer of the data to other entities and to other countries.

The child’s parents/guardians will receive results of positively identified specimens for parasites as well as those with negative results by TSET as it is the standard diagnostic test used in Ucayali central lab and in Peru to diagnose parasitic infections. Field workers will recommend to the child’s parents/guardians to have their children followed up with their healthcare provider clinic for treatment if positive to any STH or other parasites.

7.3. Regulatory Documentation

7.3.1. Regulatory Approval/Notification

This protocol and any amendment(s) must be submitted to the appropriate regulatory authorities in each respective country, if applicable. A study may not be initiated until any applicable local regulatory requirements are met.

7.3.2. Required Prestudy Documentation

The following documents must be provided to the sponsor before starting the study:

- The protocol and amendment(s), if any, signed and dated by the participating physician.
- Where appropriate, as required by local regulations, a copy of the dated and signed (or sealed, where appropriate per local regulations) written IEC/IRB approval of the protocol,

amendments, and ICF. This approval must clearly identify the specific protocol by title and number and must be signed by the chairman or authorized designee.

- Regulatory authority approval or notification, if applicable.
- Documentation of participating physician's qualifications (eg, curriculum vitae), where required.
- Any other documentation required by local regulations.

The following documents must be provided to the sponsor before enrollment of the first child:

- Signed and dated ICF and assent consent form

8. STUDY ADMINISTRATION

8.1. Changes to the Protocol

Neither the participating physician nor the sponsor will modify this protocol without a formal amendment by the sponsor. All protocol amendments must be issued by the sponsor and signed and dated by the participating physician. Protocol amendments must not be implemented without prior IEC/IRB approval, where applicable, or when the relevant competent authority has raised any grounds for non-acceptance. Documentation of amendment approval by the participating physician and IEC/IRB must be provided to the sponsor. When the change(s) involves only logistic or administrative aspects of the study, the IRB/IEC only needs to be notified.

During the course of the study, in situations where a departure from the protocol is unavoidable, the participating physician or other physician in attendance will contact the appropriate sponsor representative (see Contact Information page(s) provided separately) before implementing any departure from the protocol. In all cases, contact with the sponsor must be made as soon as possible to discuss the situation and agree on an appropriate course of action. The data recorded in the eCRF, and source documents will reflect any departure from the protocol, and the source documents will describe this departure and the circumstances requiring it.

8.2. Patient Identification and Enrollment

The participating physician agrees to complete a child identification and enrollment log to permit easy identification of each participating child during and after the study. The document will be reviewed by the sponsor contact for completeness. The child identification and enrollment log will be treated as confidential and will be filed by the participating physician in the study file. To ensure child confidentiality, no copy will be made.

The AI-DP tool will generate a QR code at the laboratory which will be placed on the Demographic Data Collection Form, Sample Processing Form, and the slides and used as an ID identifier in the eCRF.

All reports and communications relating to the study will identify participating children by an anonymized identification number and the age at initial informed consent/assent. In cases where

the child is not enrolled for data collection in the study, the date seen and age at initial informed consent/assent will be used.

8.3. Source Documentation

The primary data sources for this study will be the Demographic Data Collection Form (Attachment 2) for each child collected at their home (Visit 2) by the field worker and the Sample Processing Form (Attachment 1) for the stool sample analyses done at the laboratory by the technician. All data will be entered in the eCRF by the lab coordinator, who is an expert lab technician.

The type and level of detail of source data available for a child should be consistent with that commonly recorded by participating study personnel as a basis for routine clinical practice. Specific details required as source data for the study will be reviewed with the participating physician before the study.

The following data collected in the Demographic Data Collection Form will be recorded into the eCRF in addition to the data in the Sample Processing Form by the lab coordinator:

- Demographics (age, gender)
- School grades
- Number of SAC in the household

8.4. Case Report Form Completion

Case report forms are provided for each child in electronic format. The data capture by the local field workers and technicians will be in local language (Spanish). Data entered in the database will be in English. Accuracy of data will be the responsibility of the principal participating physician.

Electronic Data Capture will be used for this study. The study data will be transcribed by the lab coordinator from the Demographic Data Collection Form and the Sample Processing Form onto an electronic eCRF, and transmitted in a secure manner to the sponsor within the timeframe agreed upon between the sponsor and the participating study personnel. The electronic file will be considered to be the eCRF.

Worksheets may be used for the capture of some data to facilitate completion of the eCRF. Any such worksheets will become part of the child's source documentation. All data relating to the study must be recorded in eCRFs prepared by the sponsor. Data must be entered into eCRFs in English. Designated participating study personnel must complete the eCRF as soon as possible.

All eCRF entries, corrections, and alterations must be made by the lab coordinator. If necessary, queries will be generated in the eDC tool. The lab coordinator must adjust the eCRF (if applicable) and complete the query.

If corrections to an eCRF are needed after the initial entry into the eCRF, this can be done by the participating physician.

8.5. Data Quality Control

Steps to be taken to ensure the accuracy and reliability of data will include the selection of qualified study personnel, and review of data collection procedures before the study.

Guidelines for eCRF completion will be provided and reviewed with participating study personnel before the start of the study. The sponsor will review eCRFs for accuracy and completeness after transmission to the sponsor; any discrepancies will be resolved with the participating study personnel, as appropriate. After uploading of the data into the study database, they will be verified for accuracy and consistency with the data sources.

8.6. Record Retention and Archiving

The participating physician will maintain all eCRFs and all source documents that support the data collected for each child, as well as all study documents specified by the applicable regulatory requirement(s). The participating physician will take measures to prevent accidental or premature destruction of these documents.

Essential documents must be retained for at least 5 years after the completion of the final study report but will be retained for a longer period if required by applicable regulatory requirements or by an agreement with the sponsor. It is the responsibility of the sponsor to inform the participating physician as to when these documents no longer need to be retained.

If the responsible participating physician retires, relocates, or for other reasons withdraws from the responsibility of keeping the study records, custody must be transferred to a person who will accept the responsibility. The sponsor must be notified in writing of the name and address of the new custodian. Under no circumstance shall the participating physician relocate or dispose of any study documents before having obtained written approval from the sponsor.

If it becomes necessary for the sponsor or the appropriate regulatory authority to review any documentation relating to this study, the participating physician must permit access to such reports.

8.7. Study Completion/Termination

The study will be considered completed with the last stool sample collected within the study for the last child participating in the study. The final data will be sent to the sponsor (or designee) after completion of the study.

The sponsor reserves the right to terminate the study at any time for any reason at the sole discretion of the sponsor.

Reasons for the early termination of study by the sponsor or participating physician may include but are not limited to: Failure of the investigator to comply with the protocol, requirements of the local health authorities, or the sponsor's procedures; or inadequate recruitment of children.

8.8. Use of Information

All information, including but not limited to information regarding the sponsor's operations (eg, patent applications, formulas, manufacturing processes, basic scientific data, prior clinical data, formulation information) supplied by the sponsor to the participating physician and not previously published, and any data generated as a result of this study, are considered confidential and remain the sole property of the sponsor. The participating physician agrees to maintain this information in confidence and use this information only to accomplish this study, and will not use it for other purposes without the sponsor's prior written consent.

The participating physician understands that the information obtained in the study will be used by the sponsor, and thus may be disclosed as required to other clinical investigators or regulatory agencies. To permit the information obtained to be used, the participating physician is obligated to provide the sponsor with all data obtained in the study.

Any work created in connection with performance of the study and contained in the data that can benefit from copyright protection (except any publication by the participating physician as provided for below) shall be the property of the sponsor as author and owner of copyright in such work.

The results of the study will be reported in a clinical study report generated by the sponsor, which will contain data collected from all that participated in the study. The sponsor will register and/or disclose the existence of and the results of clinical studies as required by law.

8.9. Publication Policy

Consistent with Good Publication Practices and International Committee of Medical Journal Editors guidelines, the sponsor shall have the right to publish the primary (multicenter) data and information without approval from the participating physician. The participating physician has the right to publish data specific to the study after the primary data are published. If a participating physician wishes to publish information from the study, a copy of the manuscript must be provided to the sponsor for review at least 60 days before submission for publication or presentation. Expedited reviews will be arranged for abstracts, poster presentations, or other materials. If requested by the sponsor in writing, the participating physician will withhold such publication for up to an additional 60 days to allow for filing of a patent application. In the event that issues arise regarding scientific integrity or regulatory compliance, the sponsor will review these issues with the participating physician. The sponsor will not mandate modifications to scientific content and does not have the right to suppress information. For multicenter study designs and substudy approaches, where applicable, secondary results generally should not be published before the primary parameters of a study have been published. Similarly, participating physicians will recognize the integrity of a multicenter study by not submitting for publication data derived from the individual site until the combined results from the completed study have been submitted for publication, within 12 months of the availability of the final data (tables, listings, graphs), or the sponsor confirms there will be no multicenter study publication.

Authorship of publications resulting from this study will be based on the guidelines on authorship, such as those described in the Uniform Requirements for Manuscripts Submitted to Biomedical Journals, which state that the named authors must have made a significant contribution to the design of the study or analysis and interpretation of the data, provided critical review of the paper, and given final approval of the final version.

Children identifiers will not be used in the publication of results. Any work created in connection with performance of the study and contained in the data that can benefit from copyright protection (except any publication by the participating physician) shall be the property of the sponsor as author and owner of copyright in such work.

9. REFERENCES

1. Larsson J, Hedberg R. Development of machined learning models for object identification of parasite eggs using microscopy. Uppsala University June 2020.
2. World Health Organization. Prevention and control of schistosomiasis and soil-transmitted helminthiasis: report of a WHO expert committee. Geneva, 2002.
3. Saboyá MI, Catalá L, Nicholls RS, Ault SK. Update on the mapping of prevalence and intensity of infection for soil transmitted helminth infections in Latin America and the Caribbean: a call for action. PLoS Negl Trop Dis. 2013;7(9):e2419. https://doi: 10.1371/journal.pntd.0002419.
4. Hotez PJ, Bundy DAP, Brooker S, Drake L, de Silva N. Helminth infections: soil-transmitted helminth infections and schistosomiasis. In: Disease Control Priorities in Developing Countries, 2nd ed. Washington (DC): The International Bank for Reconstruction and Development / The World Bank; New York: Oxford University Press; 2006; 467-482. Available at: http://www.ncbi nlm.nih.gov/books/NBK11748/ (accessed on January 21, 2022).
5. Montressor A, Crompton DWT, Hall A, Bundy DAP, Savioli L. Guidelines for the evaluation of soil-transmitted helminthiasis and schistosomiasis at community level. Geneva:World Health Organization. 1998. Available at:http://whqlibdoc.who.int/hq/1998/WHO_CTD_SIP_98.1.pdf (accessed on January 21, 2022).
6. Smith H, Dekaminsky R, Niwas S, Soto R, Jolly P. Prevalence and intensity of infections of *Ascaris lumbricoides* and *Trichuris trichiura* and associated socio-demographic variables in four rural Honduran communities. Mem Inst Oswaldo Cruz. 2001;96(3):03-314.
7. Colston J, Saboyá MI. Soil-transmitted helminthiasis in Latin America and the Caribbean: modelling the determinants, prevalence, population at risk and costs of control at sub-national level. Geospat Health. 2013;7(2):321-340.
8. Pullan RL, Smith JL, Jasrasaria R, Brooker SJ. Global numbers of infection and disease burden of soil transmitted helminth infections in 2010. Parasit Vectors. 2014;7(1):37.
9. Hay S, Abajobir A, Abate K, et al. Global, regional, and national dis- ability-adjusted life-years (DALYs) for 333 diseases and injuries and healthy life expectancy (HALE) for 195 countries and territories, 1990–2016: a systematic analysis for the Global Burden of Disease Study 2016. Lancet. 2017;390(10100):1260–1344.
10. World Health Organization. Helminth control in school-age children: a guide for managers of control programmes. Geneva, Switzerland, 2011.
11. Funk Al, Boisson S, Clasen T, Ensink HJ. Comparison of Kato-Katz, ethyl-acetate sedimentation, and Midi Parasep in the diagnosis of *hookworm, Ascaris* and *Trichuris* infections in the context of an evaluation of rural sanitation in India. Acta Tropica. 2013;126:265–268.
12. Cools P, Vlaminck J, Albonico M, et al. Diagnostic performance of a single and duplicate Kato-Katz, Mini-FLOTAC, FECPAK^{G2} and qPCR for the detection and quantification of soil-transmitted helminths in three endemic countries. PLoS Negl Trop Dis. 2019;13(8):e0007446. https://doi.org/10.1371/journal.pntd.0007446.
13. Mupfasoni D, Mikhailov A, Mbabazi P, King J, Gyorkos TW, Montresor A. Estimation of the number of women of reproductive age in need of preventive chemotherapy for soil-transmitted helminth infections. PLoS Negl Trop Dis.2018;12(2):e000626. https://doi.org/10.1371/journal.pntd.0006269.

ATTACHMENTS

Attachment 1: Sample Processing Form

Area (A,B,C,D,E,F)	Parasitology AI Study PERU	Version 2.1
☐	June 27 2022 - Sep 30 2022	1 7 1 2 2 0 2 1<br>d d m m y y y y
FW (A,B,C,D,E,F)	Identification Number	Sample Collection Date
☐	☐☐☐<br>Correlative number	☐☐☐☐ 2 0 2 2<br>d d m m y y y y

Sample Time

Time Sample Production	Collection	Arrival Time
☐☐ : ☐☐<br>H H m m	☐☐ : ☐☐<br>H H m m	☐☐ : ☐☐<br>H H m m

Day 1 — **Scheduled Time** ☐☐ : ☐☐

Sedimentation (00 Hrs) [+ (2-5)/ ++ (6-10) / +++ (> 10)] **(MARK only: 1, 2 or 3)**

☐☐ : ☐☐
H H m m

* Hookworm= Ancylostoma /Necator

Negative	Control	
Yes	Yes	No

1	2	3	4	5	6	7	8	9	10
**Ascaris**	**Trichuris**	**Hookworm ***	**E. vermicularis**	H. nana	H. diminuta	Taenia	Fasciola	S. stercoralis	Giardia

11	Capillaria sp	12	D. pacificum	13	Trichostrongylus	14		15		16		Comments:

Slide Preparation (time for 2 slides, Slide A and B)

Start HH : mm   End HH : mm

SLIDE A - KK1.0 (30 + - 10 min)
(All Parasites Egg N°)

Factor	Stool TYPE		Start / End
8 / 12 / 24		1= Normal, formed (24)<br>2 = Pasty (12)<br>3= NON-LIQUID Diarrhea (8)	Start HH : mm End HH : mm

Negative	Control
Yes	Yes / No

1	**Ascaris**	2	**Trichuris**	3	**Hookworm**	4	**E. vermicularis**	5	H. nana	6	H. diminuta	7	Taenia	8	Fasciola
9	S. stercoralis	10	Giardia	11	Capilaria sp	12	D. pacificum	13	Trichostrongylus	14		15		16	

Day 2

SLIDE B - KK1.0 (24 H + - 4 H)
(All Parasites Egg N°)

Factor			Stool TYPE
8	12	24	

1= Normal, formed (24)

2 = Pasty (12)

3= NON-LIQUID Diarrhea (8)

Start: HH : mm   End: HH : mm

Negative	Control	
Yes	Yes	No

1 Ascaris	2 Trichuris	3 Hookworm	4 E. vermicularis	5 H. nana	6 H. diminuta	7 Taenia	8 Fasciola
9 S. stercoralis	10 Giardia	11 Capilaria sp	12 D. pacificum	13 Trichostrongylus	14	15	16

SLIDE B - KK2.0 (24 H + - 4 H)

HH : mm

QC (10 % at Random)

Day 1

Sedimentation (00 Hrs) [+ (2-5)/ ++ (6-10) / +++ (> 10)] **(MARK only: 1, 2 or 3)**

* Hookworm= Ancylostoma/Necator

__ __ : __ __
H m m

Negative
Yes

1 **Ascaris**	2 **Trichuris**	3 **Hookworm ***	4 **E. vermicularis**	5 H. nana	6 H. diminuta	7 Taenia	8 Fasciola	9 S. stercoralis	10 Giardia
+	+	+	+	+	+	+	+	+	+
11 Capillaria sp	12 D. pacificum	13 Trichostrongylus	14	15	16				
+	+	+	+	+	+				

Comments:
___

SLIDE A - KK1.0 (30 + - 10 min)
(All Parasites Egg N°)

**Factor**			**Stool TYPE**
8	12	24	

1= Normal, formed (24)

2 = Pasty (12)

3= NON- LIQUID Diarrhea (8)

__ __ : __ __
H H m m

**Negative**
Yes

1 **Ascaris**	2 **Trichuris**	3 **Hookworm**	4 **E. vermicularis**	5 H. nana	6 **H. diminuta**	7 Taenia	8 Fasciola

9	S. stercoralis	10	Giardia	11	Capilaria sp	12	D. pacificum	13	Trichostrongylus	14		15		16	

Day 2

SLIDE B - KK1.0 (24 H + - 4 H)
(All Parasites Egg N°)

Factor	Stool TYPE
8 / 12 / 24	

1= Normal, formed (24)

2 = Pasty (12)

3= NON-LIQUID Diarrhea (8)

__ __ : __ __
H H m m

Negative
Yes

1	Ascaris	2	Trichuris	3	Hookworm	4	E. vermicularis	5	H. nana	6	H. diminuta	7	Taenia	8	Fasciola
9	S. stercoralis	10	Giardia	11	Capilaria sp	12	D. pacificum	13	Trichostrongylus	14		15		16	

Supervised:

Initials

__ __ __ __ 2 0 2 2
d d m m y y y y

Initials

**Digitized:**	[ ][ ][ ]	[ ][ ][ ][ ] 2 0 2 2<br>d d m m y y y y

Attachment 2: Demographic Data Collection Form

Parasitology AI Study Peru

Area (A,B,C,D,E,F)		Field Worker (A,B,C,D,E,F)		QRC ID Code #	

Information Collection Date	_____ / _____ / 2022 (use format: dd / mm / 2022)

Child full Name			
Age	Gender	School Grade	Are there OTHER children between 5-14 years in the house?
	M F<br>☐ ☐	☐ Pre-school ☐ Primary<br>☐ Secondary ☐ Not in School	☐ no other child at this age<br>☐ 1 ☐ 2 ☐ 3 or more

Obtained the *Parent/Legal Guardian Information and Permission Form*?	☐ Yes	☐ No. Why? ____________
Obtained the *Assent Form* from the child?	☐ Yes	☐ No. Why? ____________

Inclusion Criteria	Yes	No
Male or female aged 5 to 14 years of age, inclusive, as this group is at high risk for STH morbidity		
Child is otherwise in healthy condition as stated by their parents		
Parent(s)/guardian(s) of children understand the purpose and procedures required for the study and that they are willing to have their child participate in the study		
Child of 8 years or older understand the purpose of the study and procedures required for the study, and are willing to participate in the study		
Child is able to provide an adequate stool sample as specified on stool collection container		
Parent/Guardian and child speak Spanish		
**Exclusion Criteria**	**Yes**	**No**
Child has active diarrhea (defined as the passage of 3 or more loose or liquid stools per day) at baseline		
Child has an acute medical condition or is experiencing a severe concurrent medical condition		
Child has received anthelmintic treatment within 90 days prior to study entry		
Child is not able to provide an adequate stool sample as specified on stool collection container		

Is the child eligible to be enrolled in the study? ☐ Yes ☐ No

____ ____________

Field worker name and signature **Date**

PARTICIPATING PHYSICIAN AGREEMENT

VERMOX (mebendazole)

Protocol 108329HEL4001

PARTICIPATING PHYSICIAN AGREEMENT

I have read this protocol and agree that it contains all necessary details for carrying out this study. I will conduct the study as outlined herein and will complete the study within the time designated.

I will provide copies of the protocol and all pertinent information to all individuals responsible to me who assist in the conduct of this study. I will discuss this material with them to ensure that they are fully informed regarding the conduct of the study and the obligations of confidentiality.

Principal Participating Physician:

Name (typed or printed): PPD [redacted]

Institution and Address: [redacted]

Telephone Number: [redacted]

Signature: [redacted] Date: 26 / 03 / 2022

(Day Month Year)

Sponsor's Responsible M[illegible]

Name (typed or printed): PPD [redacted]

Institution: Janssen Research & Development, LLC

Signature: ______________________ Date: ______________

(Day Month Year)

Note: If the address or telephone number of the investigator changes during the course of the study, written notification will be provided by the investigator to the sponsor; a protocol amendment will not be required.

LAST PAGE

CONFIDENTIAL – FOIA Exemptions Apply in U.S. 42

Status: Approved, Date: 26 March 2022
