# Supplementary material for: Artificial intelligence-based digital pathology for the detection and quantification of soil-transmitted helminths eggs
Source: PLoS Negl Trop Dis. 2024 Sep 30;18(9):e0012492. doi: 10.1371/journal.pntd.0012492 (PMC11488745; doi:10.1371/journal.pntd.0012492)
Supplement: S2 Info — (DOCX) [file pntd.0012492.s002.docx]

**S1 Info**

### AI Egg Verification Process

The verification process for artificial intelligence (AI)-based egg detections from the KK2.0 scanner-collected images includes object detections, two rounds of verification, and a conflict resolution process (**Fig S1**).

The generation of the AI-based object detections include ‘*Ascaris’*, ‘*Trichuris’*, ‘hookworm’, ‘*Schistosoma’*, ‘unsure object’ (when the detected object may be considered positive for more than one egg type), and ‘not an egg’ (when the confidence of the detection is low).

Verification of all AI-based object detections is done by a single verifier, in which detected eggs are presented to the verifier one page at a time. Each page consists of 9 detections in a 3x3 grid, with objects of the same label. For each visible object, the verifier may decide to:

- Accept the AI detection, verifying the object type,
- Reject the AI detection, setting the object label to ‘not an egg’,
- Change the AI detection to another egg type
- Mark the AI detection as ‘unsure.’

A second-round of verification of objects (including eggs and objects not considered eggs) is performed to capture any mistake that the first verifier may have performed. The second-round verifier(s) follow the identical methods as the first-round verifier(s), but instead of looking at objects with labels as assigned by the AI model, they look observe the label assigned to objects following the first-round verification. The conflict resolution process (**Fig S2**), is used to reach a final determination of object labels, as follows:

1. If first verifier and the second verifier agree on an object label, that label will be considered in the result output.
2. If the first verifier and the second verifier disagree on an object label, then a third verifier is assigned to review the object.
3. If the third verifier agrees with verifier 1 or verifier 2, then the resulting output will be that made by verifier 3.
4. If all verifiers disagree, then in the resulting output, the object label would be ‘unsure’.

Fig S1. The AI Egg Verification Process


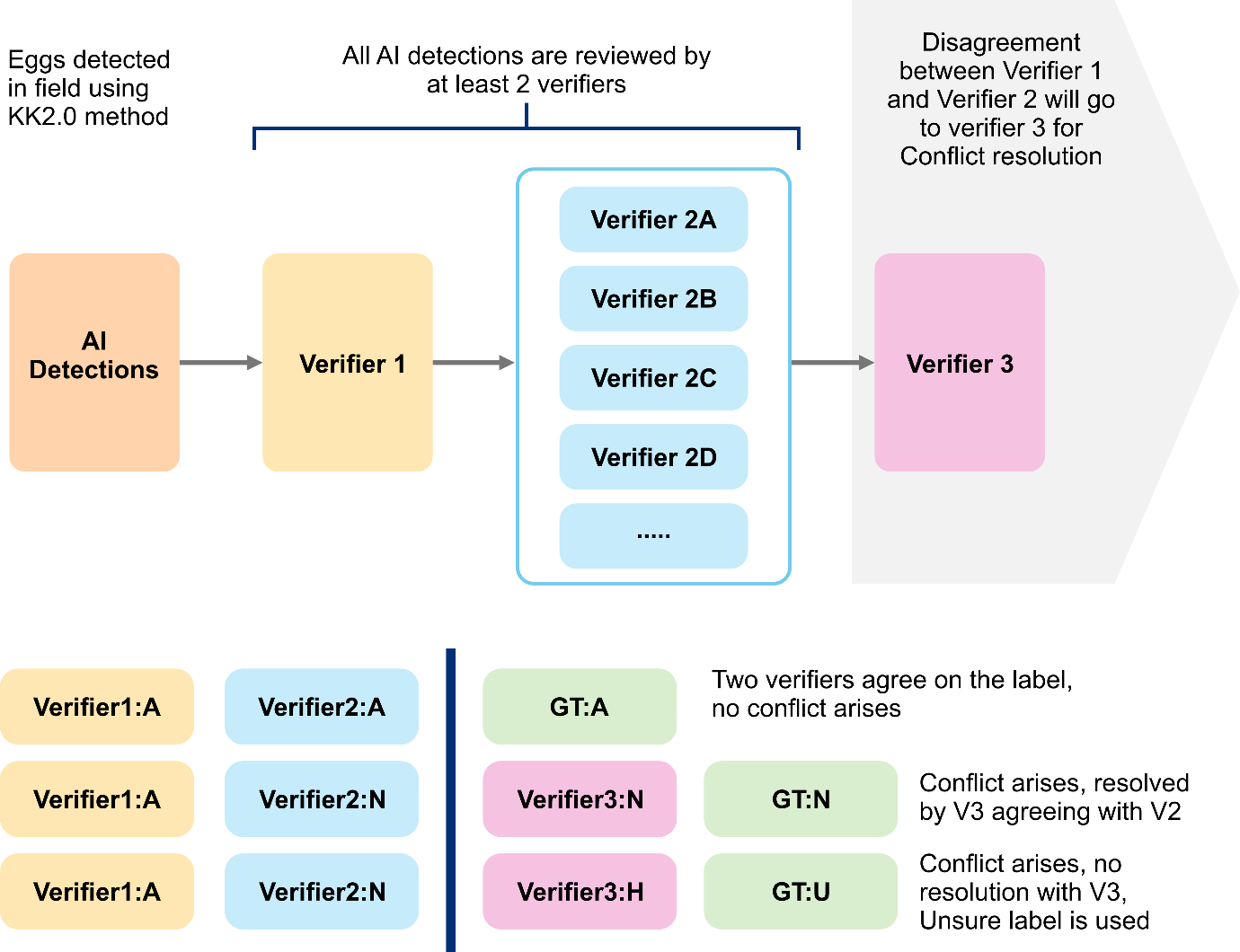


AI, artificial intelligence; KK2.0, artificial intelligence digital pathology Kato Katz method

Fig S2. Conflict Resolution Examples

Conflicts are never to be resolved by the verifiers who created the conflict. A third verifier is always called.

A, Ascaris; GT, ground true; H, hookworm; N, not an egg; U, unsure; V, verifier.

This AI process ensures accurate and reliable verification of egg detections using an AI-based object detection method, employing multiple rounds of verification and conflict resolution to eliminate errors and uncertainties, resulting in high-quality data output.
