# Supplementary material for: Artificial intelligence-based digital pathology for the detection and quantification of soil-transmitted helminths eggs
Source: PLoS Negl Trop Dis. 2024 Sep 30;18(9):e0012492. doi: 10.1371/journal.pntd.0012492 (PMC11488745; doi:10.1371/journal.pntd.0012492)
Supplement: S1 Table — (DOCX) [file pntd.0012492.s003.docx]

**S1 Table.** **Demographics and baseline characteristics (Analysis set: all enrolled)**

| Parameter | N=510 |
| --- | --- |
| Age, Mean (SD), years | 9.7 (2.80) |
| Gender, Girls, n (%) | 255 (50.0) |
| Educational Status, n (%) | |
| Initial/Preprimary | 83 (16.3) |
| Primary | 338 (66.3) |
| Secondary | 80 (15.7) |
| Not in School | 9.0 (1.8) |
| Other children between 5-14 years in the house | |
| 1 | 178 (34.9) |
| 2 | 102 (20.0) |
| 3 or more | 75 (14.7) |
| No other child at this age | 155 (30.4) |
| SD, standard deviation |  |
