# Supplementary material for: Artificial intelligence-based digital pathology for the detection and quantification of soil-transmitted helminths eggs
Source: PLoS Negl Trop Dis. 2024 Sep 30;18(9):e0012492. doi: 10.1371/journal.pntd.0012492 (PMC11488745; doi:10.1371/journal.pntd.0012492)
Supplement: S2 Table — (DOCX) [file pntd.0012492.s004.docx]

**S2 Table. EPG by diagnostic method, STH species, and timepoint - positive samples; evaluable**

| STH Species | Timepoint | KK1.0 | | | KK2.0 | | | *p*-value |  |
| --- | --- | --- | --- | --- | --- | --- | --- | --- | --- |
|  |  | **N** | **Mean (SD)** | **95% CI** | **N** | **Mean (SD)** | **95% CI** |  |  |
| *Ascaris lumbricoides* | 30-minute | 191 | 23193.5 (60335.4) | (15534.3, 32504.4) | 241 | 15435.4 (34464.2) | (11390.1, 20035.2) | 0.021 |  |
|  | 24-hour | 186 | 18485.4 (29320.6) | (14453.8, 22890.0) | 213 | 16448.5 (35547.6) | (11993.2, 21560.1) | 0.295 |  |
| *Trichuris trichiura* | 30-minute | 117 | 1217.6 (3599.3) | (646.7, 1936.3) | 118 | 1512.0 (6068.7) | (598.3, 2749.8) | 0.431 |  |
|  | 24-hour | 136 | 1916.8 (8306.5) | (765.2, 3528.3) | 112 | 984.0 (3874.6) | (394.6, 1806.7) | 0.167 |  |
| Hookworms | 30-minute | 13 | 282.5 (547.1) | (48.0, 620.7) | 9 | 197.3 (353.7) | (32.0, 471.0) | 0.692 |  |
| 95% confidence interval (95% CI) is based on bootstrapping. A permutation test was employed for comparison of eggs per gram of stool (EPG) between different diagnosis methods based on absolute mean difference. Hookworm samples are excluded from analysis if the time between slide preparation end time and slide reading start time is outside the range of 20 to 80 minutes (inclusive).  KK1.0, traditional Kato-Katz method; KK2.0, artificial intelligence digital pathology Kato-Katz method; SD, standard deviation; STH, soil-transmitted helminth. | | | | | | | | |  |
