# Supplementary material for: Artificial intelligence-based digital pathology for the detection and quantification of soil-transmitted helminths eggs
Source: PLoS Negl Trop Dis. 2024 Sep 30;18(9):e0012492. doi: 10.1371/journal.pntd.0012492 (PMC11488745; doi:10.1371/journal.pntd.0012492)
Supplement: S3 Table — (DOCX) [file pntd.0012492.s005.docx]

**S3 Table.** **Prevalence of STH infections by diagnostics method, STH species and month after MDA**

|  | **KK1.0** | **KK2.0** | **TSET** |
| --- | --- | --- | --- |
| **N** | **508** | **508** | **508** |
| *Ascaris lumbricoides*, n/N (%) | | | |
| August | 81/166 (48.8) | 111/166 (66.9) | 67/166 (40.4) |
| September | 92/246 (37.4) | 131/246 (53.3) | 80/246 (32.5) |
| October | 45/96 (46.9) | 58/96 (60.4) | 46/96 (47.9) |
| *Trichuris trichiura*, n/N (%) | | | |
| August | 64/166 (38.6) | 45/166 (27.1) | 47/166 (28.3) |
| September | 57/246 (23.2) | 58/246 (23.6) | 44/246 (17.9) |
| October | 32/96 (33.3) | 30/96 (31.3) | 28/96 (29.2) |
| Hookworms, n/N (%) | | | |
| August | 8/164 (4.9) | 3/150 (2.0) | 8/166 (4.8) |
| September | 5/246 (2.0) | 5/231 (2.2) | 7/246 (2.8) |
| October | 0/96 (0.0) | 1/91 (1.1) | 1/96 (1.0) |
| If any reading of a sample is positive for a certain STH species, the sample is considered positive for that species for that diagnostics method. A sample is assigned to the month in which the sample is collected.  KK1.0, traditional Kato Katz method; KK2.0, artificial intelligence digital pathology Kato Katz method; MDA, mass drug administration; STH, soil-transmitted helminths; TSET, tube spontaneous sedimentation technique**.** | | | |
